# Post-Translational Protein Deimination Signatures in Plasma and Plasma EVs of Reindeer (*Rangifer tarandus*)

**DOI:** 10.3390/biology10030222

**Published:** 2021-03-13

**Authors:** Stefania D’Alessio, Stefanía Thorgeirsdóttir, Igor Kraev, Karl Skírnisson, Sigrun Lange

**Affiliations:** 1 Tissue Architecture and Regeneration Research Group, School of Life Sciences, University of Westminster, London W1W 6UW, UK; w1650366@my.westminster.ac.uk; 2 Institute for Experimental Pathology at Keldur, University of Iceland, Keldnavegur 3, 112 Reykjavik, Iceland; stef@hi.is (S.T.); karlsk@hi.is (K.S.); 3 Electron Microscopy Suite, Faculty of Science, Technology, Engineering and Mathematics, Open University, Milton Keynes MK7 6AA, UK; igor.kraev@open.ac.uk

**Keywords:** protein deimination/citrullination, peptidylarginine deiminase (PAD), extracellular vesicles (EVs), reindeer (*Rangifer tarandus*), immunity, metabolism, gene regulation, prion disease, zoonosis

## Abstract

**Simple Summary:**

Reindeer are an important wild and domesticated species of the Arctic, Northern Europe, Siberia and North America. As reindeer have developed various strategies to adapt to extreme environments, this makes them an interesting species for studies into diversity of immune and metabolic functions in the animal kingdom. Importantly, while reindeer carry natural infections caused by viruses (including coronaviruses), bacteria and parasites, they can also act as carriers for transmitting such diseases to other animals and humans, so called zoonosis. Reindeer are also affected by chronic wasting disease, a neuronal disease caused by prions, similar to scrapie in sheep, mad cows disease in cattle and Creutzfeldt-Jakob disease in humans. The current study assessed a specific protein modification called deimination/citrullination, which can change how proteins function and allow them to take on different roles in health and disease processes. Profiling of deiminated proteins in reindeer showed that many important pathways for immune defenses, prion diseases and metabolism are enriched in deiminated proteins, both in plasma, as well as in plasma extracellular vesicles. This study provides a platform for the development of novel biomarkers to assess wild life health status and factors relating to zoonotic disease.

**Abstract:**

The reindeer (caribou) *Rangifer tarandus* is a Cervidae in the order Artiodactyla. Reindeer are sedentary and migratory populations with circumpolar distribution in the Arctic, Northern Europe, Siberia and North America. Reindeer are an important wild and domesticated species, and have developed various adaptive strategies to extreme environments. Importantly, deer have also been identified to be putative zoonotic carriers, including for parasites, prions and coronavirus. Therefore, novel insights into immune-related markers are of considerable interest. Peptidylarginine deiminases (PADs) are a phylogenetically conserved enzyme family which causes post-translational protein deimination by converting arginine into citrulline in target proteins. This affects protein function in health and disease. Extracellular vesicles (EVs) participate in cellular communication, in physiological and pathological processes, via transfer of cargo material, and their release is partly regulated by PADs. This study assessed deiminated protein and EV profile signatures in plasma from sixteen healthy wild female reindeer, collected in Iceland during screening for parasites and chronic wasting disease. Reindeer plasma EV profiles showed a poly-dispersed distribution from 30 to 400 nm and were positive for phylogenetically conserved EV-specific markers. Deiminated proteins were isolated from whole plasma and plasma EVs, identified by proteomic analysis and protein interaction networks assessed by KEGG and GO analysis. This revealed a large number of deimination-enriched pathways for immunity and metabolism, with some differences between whole plasma and EVs. While shared KEGG pathways for whole plasma and plasma EVs included complement and coagulation pathways, KEGG pathways specific for EVs were for protein digestion and absorption, platelet activation, amoebiasis, the AGE–RAGE signaling pathway in diabetic complications, ECM receptor interaction, the relaxin signaling pathway and the estrogen signaling pathway. KEGG pathways specific for whole plasma were pertussis, ferroptosis, SLE, thyroid hormone synthesis, phagosome, *Staphylococcus aureus* infection, vitamin digestion and absorption, and prion disease. Further differences were also found between molecular function and biological processes GO pathways when comparing functional STRING networks for deiminated proteins in EVs, compared with deiminated proteins in whole plasma. This study highlights deiminated proteins and EVs as candidate biomarkers for reindeer health and may provide information on regulation of immune pathways in physiological and pathological processes, including neurodegenerative (prion) disease and zoonosis.

## 1. Introduction

The reindeer (*Rangifer tarandus*), also known as the caribou in North America, is a mammal of the order Artiodactyla, family Cervidae, and has a circumpolar distribution. Reindeer play an important role in economy, society, culture and ecological values among populations of Eurasia and were fundamental for the colonization of the northern part of Eurasia. They have been a source of food among indigenous culture and were sporadically used for transportation, and therefore can be considered as a semi-domesticated species [1]. Reindeer are adapted to extreme environments throughout their evolution, exhibiting distinctive and unique biological characteristics relating to fat metabolism processes, changes to their internal biological clock, limited heat loss and low resting metabolic rate. However, the underlying molecular and genetic basis for these traits remains largely unknown [1,2]. The IUCN Red List of Threatened Species (2016) has classified reindeer as a vulnerable species due to a decline of individuals, possibly attributed to habitat shift and/or their susceptibility to chronic wasting disease (CWD), a fatal neurodegenerative disorder [3,4]. Importantly also, *R. tarandus* may play roles in various zoonotic diseases, including parasitic, bacterial and viral ones [5,6,7,8], and deer have furthermore been recently identified to be new reservoir hosts for SARS-CoV-2 [9].

While the reindeer genome has been sequenced [10], and genetic diversity and mitochondrial DNA have furthermore been studied [11], no studies have hitherto been performed into mechanisms relating to post-translational modifications such as deimination, which is caused by peptidylarginine deiminases (PADs). Furthermore, while research on extracellular vesicles (EVs) is a major field in relation to biomarker discovery in human pathologies, and recent comparatives studies have highlighted their value in a range of wild, domestic and commercially valuable land and aquatic animals throughout the phylogeny tree [12,13,14,15,16,17,18,19,20,21,22,23,24,25], the field is still in its infancy in relation to studies and biomarker development in wild animals.

Peptidylarginine deiminases (PADs) are a phylogenetically conserved calcium-dependent family of enzymes. PADs convert arginine into citrulline in an irreversible manner, leading to post-translational modification (citrullination/deimination) in numerous target proteins of cytoplasmic, nuclear and mitochondrial origin [17,26,27,28,29,30]. Deimination causes structural protein changes which can affect protein function and consequently downstream protein–protein interactions. Deimination can, among other, contribute to neo-epitope generation, which results in inflammatory responses, as well as affect gene regulation and neutrophil extracellular trap formation (NETosis) via deimination of histones [31,32,33,34,35]. As post-translational changes contribute to protein moonlighting, which allows one protein to exhibit different functions within one polypeptide chain [36], deimination may facilitate such functional diversity of proteins in health and disease.

In mammals, five PAD isozymes are known, while lower in the phylogeny tree, there is less diversification of PADs, with three PAD isozymes described in birds and reptiles, but only one PAD form in fish [17,18,26,28,29,37]. Furthermore, PAD homologues, also referred to as arginine deiminases (ADI) [38] have been described in parasites [39], bacteria [40,41] and fungi [42]. This places PADs as important proteins both in host immune defenses, as well as in host–pathogen interactions.

A majority of studies on PADs and downstream deimination have related to human pathological mechanisms, but recently a comparative body of research has focused on identifying putative roles for PADs in physiological and immunological pathways in a wide range of taxa throughout the phylogenetic tree, including land and sea mammals, reptiles, birds, bony and cartilaginous fish, Mollusca and Crustacea [16,17,18,19,20,21,22,23,24,25,28,29]. PADs have furthermore been identified to have roles in mucosal, innate and adaptive immunity in a range of taxa [17,18,19,20,25,28,29,43,44]. Importantly, PADs have also been identified as important players in infection and anti-pathogenic responses, including anti-viral [45,46] and anti-parasitic ones [39], as well as in anti-bacterial mechanisms [40,41]. Furthermore, as roles for PADs in EV release and deiminated protein cargo in EVs in health and disease, in diverse taxa and comparative animal models of disease, are gaining increased attention, investigations into reindeer may be of some interest.

EV biogenesis and regulation of EV release from cells has been shown to be regulated by PADs to some extent, and as this has been identified in a range of taxa, it appears to be a phylogenetically conserved function [39,41,47,48,49,50]. EVs participate in cellular communication and can be isolated from many body fluids, including serum, plasma, saliva and urine. EVs play physiological and pathological roles via transfer of cargo proteins and genetic material, including in inflammatory responses, in infection and host–pathogen interactions [34,39,51,52,53,54,55]. As EVs carry information from their cells of origin, their cargo signatures are usable biomarkers [56,57]. Currently, as relatively few studies on EVs have been conducted in wild animals [14,15,25] this is a pioneering and promising field for novel biomarker discovery and development.

As, to date, neither PADs and associated deimination nor EVs have been assessed in reindeer, the current study aimed at profiling protein deimination in plasma and plasma EVs for assessment of regulation on protein networks and identification of putative biomarkers to gain new insights into both immune system and metabolic adaptations of reindeer. This study may further current understanding of the roles for post-translational modifications in functional diversification of conserved proteins throughout phylogeny.

## 2. Materials and Methods

### 2.1. Plasma Sampling

Blood samples were taken from sixteen individual female reindeer (*R. tarandus*)—average age approximately 7 years (range 1.5–12 years old)—sampled in Iceland. The sampling was part of research dealing with general health of Icelandic reindeer with specific emphasis on chronic wasting disease (CWD) and presence of parasites. Sample collection was in accordance with Icelandic laws and regulations on sampling from wild animals (64/1994) and licenses of the Institute for Experimental Pathology at Keldur, University of Iceland (number #0001 kt-650269—4549), approved by the central animal ethics committee in Iceland (Icelandic Food Regulation Authority, MAST Matvælastofnun). The plasma was isolated according to standard procedures from EDTA blood samples. Brain samples from the same animals were screened for presence of prion disease by ELISA (TeSeE^®^, Bio-Rad, UK) following standard procedures at the Institute for Experimental Pathology at Keldur, and the animals were confirmed to be disease free and healthy. Plasma was aliquoted at 250 µL and stored at −80 °C until further use for the individual experiments.

### 2.2. Isolation of Extracellular Vesicles and Nanoparticle Tracking Analysis (NTA)

Reindeer plasma EVs were prepared from the individual plasma (thawed on ice) of the sixteen animals using sequential centrifugation and ultracentrifugation. Procedures were carried out according to our previously standardized and described protocols [18,23,43], also following recommendations of MISEV2018 (the minimal information for studies of extracellular vesicles 2018) [58]. For each individual plasma EV preparation, 100 μL of reindeer plasma was diluted 1:5 in Dulbecco’s PBS (DPBS, ultrafiltered using a 0.22 μm filter, before use). This was then centrifuged for 20 min at 4000× *g* at 4 °C, to remove apoptotic bodies and aggregates. Supernatants were then collected and ultra-centrifuged at 100,000× *g* at 4 °C for 1 h. This resulted in EV-enriched pellets, which were resuspended each in 500 µL DPBS and thereafter ultra-centrifuged again for 1 h at 100,000× *g*, at 4 °C. The final resulting EV pellets were resuspended each in 100 µL of DPBS. The EVs were kept frozen at −80 °C until used in the procedures described below (all assessments were performed with EV preparations that had not been frozen for longer than 1 week). Plasma EV size distribution profiles were generated and EVs were quantified using nanoparticle tracking analysis (NTA), based on Brownian motion of particles in suspension, and carried out using the NanoSight NS300 system (Malvern Panalytical Ltd., Malvern, UK). Prior to application on the NanoSight, the EV samples were diluted 1/100 in DPBS (10 μL of EV preparation diluted in 990 μL of DPBS). The diluted EV samples were applied to the NanoSight NS300 (Malvern Panalytical, UK), recording four repetitive reads, 60 sec each. Particle numbers per frame were 40 to 60, camera settings were at level 12 for recording and for post-analysis the threshold was set at 3. Replicate histograms were generated from these videos using the NanoSight software 3.0 (Malvern), representing mean and confidence intervals of the four recordings for each sample.

### 2.3. Transmission Electron Microscopy (TEM)

Plasma EVs were assessed for morphology using TEM, using a pool of plasma EVs from five animals. The procedure was similar as previously described [16,20]. Following thawing of isolated EV pellets (stored frozen for 1 week before imaging), the EVs were resuspended in 100 mM sodium cacodylate buffer (pH 7.4). One drop (~3–5 μL) of the EV suspension was placed onto a grid which held a carbon support film which had been previously glow-discharged. Following partial drying of the EV suspension, the sample was fixed for 1 min at room temperature by placing the grid onto a drop of a fixative solution (2.5% glutaraldehyde in 100 mM sodium cacodylate buffer (pH 7.0)). The grid was applied to the surface of three drops of distilled water for washing of the EV sample, removing excess water using a filter paper. The EVs were then stained for 1 min with 2% aqueous Uranyl Acetate (Sigma-Aldrich), removing excess stain with a filter paper and air drying the grid. TEM imaging of EVs was carried out with a JEOL JEM 1400 transmission electron microscope (JEOL, Tokyo, Japan), which was operated at 80 kV, using a magnification of 30,000× to 60,000×. Recording of digital images was performed with an AMT XR60 CCD camera (Deben, UK).

### 2.4. Isolation of Deiminated Proteins Using F95 Enrichment

Total deiminated proteins were isolated from reindeer plasma and plasma EVs using the F95 pan-deimination antibody (MABN328, Merck, UK) and the Catch and Release^®^v2.0 immunoprecipitation kit (Merck), according to previously described methods in a range of taxa [16,18,20,23,43]. The F95-antibody specifically detects proteins modified by citrullination and has been developed against a deca-citrullinated peptide [59]. Pools of plasma from five individual animals (5 × 20 μL) and, correspondingly, EV isolates from the same five individual animals (5 x 20 μL EVs) were used for F95 enrichment, which was performed at 4 °C overnight, using a rotating platform. Elution of deiminated (F95-bound) proteins from the columns was performed with the elution buffer provided with the immunoprecipitation kit and according to the manufacturer’s instructions (Merck), and the protein eluate was thereafter diluted 1:1 in 2× Laemmli sample buffer (BioRad, Watford, UK). Samples were kept frozen at −20 °C until further use for SDS-PAGE analysis, Western blotting and in-gel digestion for LC–MS/MS analysis, as described below.

### 2.5. Western Blotting Analysis

For Western blotting, SDS-PAGE was carried out on plasma, as well as plasma EV samples. All samples were diluted 1:1 in denaturing 2× Laemmli sample buffer (containing 5% beta-mercaptoethanol, BioRad, UK) and heated for 5 min at 100 °C. Protein separation was carried out using 4–20% gradient TGX gels (BioRad UK), followed by Western blotting at 165 V for 1 h on a Trans-Blot^®^ SD semi-dry transfer cell (BioRad, UK). Membranes were stained with PonceauS (Sigma, UK) to assess even protein transfer and then blocked with 5% bovine serum albumin (BSA, Sigma, UK) in Tris-buffered saline (TBS) containing 0.1% Tween20 (BioRad, UK; TBS-T) for 1 h at room temperature. Primary antibody incubation was carried out overnight at 4 °C on a shaking platform using the following antibodies for reindeer plasma: F95 pan-deimination antibody (MABN328, Merck; diluted 1/1000 in TBS-T) and anti-human PAD2 (ab50257, Abcam, diluted 1/1000 in TBS-T), PAD3 (ab50246, diluted 1/1000 in TBS-T) and PAD4 (ab50332, diluted 1/1000 in TBS-T) antibodies, for detection of putative PAD protein homologues. PAD2 is considered the most conserved PAD isozyme and the anti-human PAD2 antibody was previously shown to cross-react with PADs across taxa [17,18,19,20,21,22,23,24,25,28,29,60,61], while both the PAD3 and PAD4 antibodies have also been found to cross react with other species, including bird, reptile and bovine [18,20,60]. EV-cargo was also assessed for PAD2, PAD3 and PAD4 as well as deiminated proteins (F95). For characterization of reindeer plasma EVs, the EV-markers CD63 (ab216130, Abcam, UK) and Flotillin-1 (ab41927); diluted 1/1000 in TBS-T) were used, and both have previously been shown to cross-react with EVs from other taxa, besides human. Following primary antibody incubation overnight at 4 °C, the nitrocellulose membranes were washed at RT in TBS-T for 3 × 10 min and thereafter incubated with HRP-conjugated secondary antibodies (anti-rabbit IgG, BioRad; or anti-mouse IgM, BioRad, respectively, diluted 1/3000 in TBS-T), for 1 h at RT. The membranes were then washed for 4 × 10 min TBS-T, followed by one wash in TBS without Tween20 and digitally visualized, using enhanced chemiluminescence (ECL, Amersham, UK) in conjunction with the UVP BioDoc-ITTM System (Thermo Fisher Scientific, Dartford, UK).

### 2.6. Silver Staining

SDS-PAGE (using 4–20% gradient TGX gels, BioRad, UK) was carried out under reducing conditions for the F95-enriched protein eluates from both whole plasma and plasma EVs. The gels were then silver stained using the BioRad Silver Stain Plus Kit (1610449, BioRad, UK), according to the manufacturer’s instructions.

### 2.7. Liquid Chromatography with Tandem Mass Spectrometry (LC–MS/MS) Analysis of Deiminated Protein Candidates

Liquid chromatography with tandem mass spectrometry (LC–MS/MS) was carried out to identify deiminated proteins from reindeer plasma and plasma EVs (pool of *n* = 5 deer for plasma as well as plasma EVs, using the isolates from the same animals), according to previously described methods in other taxa [17,19,20]. Before LC–MS/MS analysis, the F95-enriched protein preparations (diluted 1:1 in 2× Laemmli buffer and boiled for 5 min at 100 °C) were run 0.5 cm into a 12% TGX gel (BioRad, UK). The concentrated protein band (containing the F95 eluate) was excized, trypsin digested and subjected to proteomic analysis using a Dionex Ultimate 3000 RSLC nanoUPLC (Thermo Fisher Scientific Inc., Waltham, MA, USA) system in conjunction with a QExactive Orbitrap mass spectrometer (Thermo Fisher Scientific Inc, Waltham, MA, USA). Peptide separation was performed using reverse-phase chromatography (flow rate 300 nL/min) and a Thermo Scientific reverse-phase nano Easy-spray column (Thermo Scientific PepMap C18, 2 µm particle size, 100 A pore size, 75 µm i.d. × 50 cm length). Peptides were loaded onto a pre-column (Thermo Scientific PepMap 100 C18, 5 µm particle size, 100 A pore size, 300 µm i.d. × 5 mm length) from the Ultimate 3000 autosampler (0.1% formic acid for 3 min, flow rate 10 µL/min). Thereafter, peptides were eluted from the pre-column onto the analytical column. The linear gradient employed was 2–40% solvent B (80% acetonitrile, 20% water + 0.1% formic acid) for 30 min. An Easy-Spray source (Thermo Fisher Scientific Inc.) was used to spray the LC eluant into the mass spectrometer. An Orbitrap mass analyzer (set at a resolution of 70,000) was used to measure all *m*/*z* values of eluting ions, scanned between *m*/*z* 380 and 1500. Fragment ions were automatically isolated and generated using data-dependent scans (Top 20) by higher-energy collisional dissociation (HCD, NCE: 25%) in the HCD collision cell. The resulting fragment ions were measured using the Orbitrap analyzer set at a resolution of 17,500. Singly charged ions and ions with unassigned charge states were excluded from selection for MS/MS, employing a dynamic exclusion window of 20 s. The data were processed post-run, using Protein Discoverer (version 2.1., Thermo Scientific). All MS/MS data were converted to mgf files. The files were submitted to the Mascot search algorithm (Matrix Science, London, UK) to identify deiminated protein hits. Search was conducted against a common UniProt database against Artiodactyla (CCP_ Artiodactyla Artiodactyla_20201013; 840,112 sequences; 473,198,619 residues). An additional search was conducted against a common contaminant database (cRAP 20190401; 125 sequences; 41,129 residues). The fragment and peptide mass tolerances were set to 0.1 Da and 20 ppm, respectively. The significance threshold value was set at of *p* < 0.05 and a peptide cut-off score of 46 for the common Artiodactyla database (carried out by Cambridge Proteomics, Cambridge, UK).

### 2.8. Protein–Protein Interaction Network Analysis

To predict and identify putative protein–protein interaction networks associated with the deiminated proteins from reindeer plasma and plasma EVs, STRING analysis (Search Tool for the Retrieval of Interacting Genes/Proteins; https://string-db.org/) was performed. Protein networks were generated based on protein names and applying the function of “search multiple proteins” in STRING (https://string-db.org/) using the Artiodactyla protein database. For a representative choice of a Artidoactyla protein database, *Bos taurus* was selected, as no species-specific *Rangifer tarandus* protein database is available in STRING, and within Artiodactyla the highest protein hit match was found with *Bos taurus*. Parameters applied in STRING were “basic settings” and “medium confidence”. Nodes are connected with color lines which represent the following evidence-based interactions for the network edges: “known interactions” (these are based on experimentally determined curated databases), “predicted interactions” (these are based on gene neighborhood, gene co-occurrence, gene fusion, via text mining, protein homology or co-expression). Gene ontology (GO) and KEGG (Kyoto Encyclopedia of Genes and Genomes) pathways for the deiminated protein networks were furthermore assessed in STRING and are highlighted by color coding (for each network analysis figure, please see the corresponding color code key included for the individual nodes and connective lines).

### 2.9. Neighbor-Joining Tree Construction for PADs from Deer

To reconstruct a phylogeny tree for Artiodactyla PADs, protein sequences of known and previously reported PAD isozymes from several deer were compared with other mammals. No PAD protein sequences have been reported for reindeer in open protein databases. For reconstruction of a neighbor joining tree, PAD sequences were therefore used from white-tailed deer (*Odocoileus virginianus texanus*), red deer (*Cervus elaphus hippelaphus*) and cow (*Bos taurus*) and compared with human PADs. The following sequences were used for the neighbor joining tree construction (using Clustal Omega https://www.ebi.ac.uk/Tools/msa/clustalo/): *Odocoileus virginianus texanus* PAD1 (XP_020733655.1), PAD2 (XP_020733656.1), PAD3 (XP_020733658.1), PAD4 (XP_020754850.1) and PAD6 (XP_020754849.1) isozymes; *Bos taurus* PAD1 (NP_001094742.1), PAD2 (NP_001098922.1), PAD3 (XP_010800991.1), PAD4 (NP_001179102.1), and PAD6 (XP_002685843.1) isozymes; *Cervus elaphus hippelaphus* PAD1 (OWK12974.1), PAD4 (OWK12644.1) isozymes, human (*Homo sapiens*) PAD1 (NP_037490.2), PAD2 (NP_031391.2), PAD3 (NP_057317.2), PAD4 (NP_036519.2) and PAD6 (NP_997304.3). A neighbor-joining phylogeny tree was constructed following sequence alignment, and homology of deer PADs between human and deer was determined by percent identity matrix, using Clustal Omega (https://www.ebi.ac.uk/Tools/msa/clustalo/).

### 2.10. Statistical Analysis

Generation of NTA curves was carried out using the Nanosight 3.0 software (Malvern, UK). The NTA curves show mean (black line) and standard error of mean (SEM), and the confidence intervals are indicated (red line). Protein–protein interaction networks were generating using STRING (https://string-db.org/), applying basic settings and medium confidence. Significance was considered as *p* ≤ 0.05.

## 3. Results

### 3.1. Characterization of Reindeer Plasma EVs

**Figure 1:**
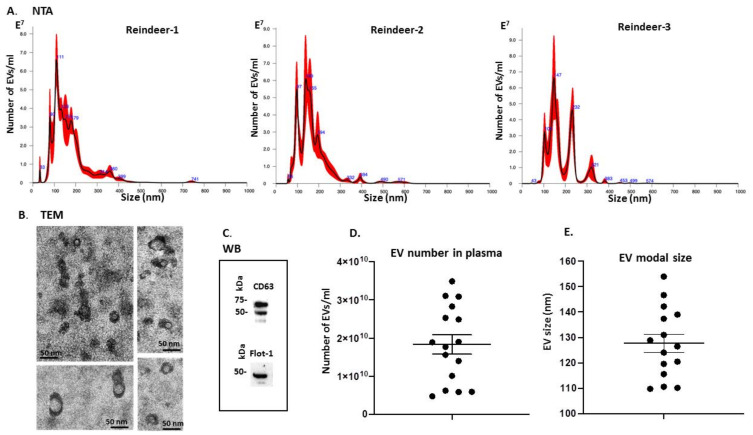
Plasma EV profiling of *Rangifer tarandus*. (**A**) Nanoparticle tracking analysis (NTA) showing a poly-dispersed distribution of reindeer plasma EVs in the size range of 30–400 nm, from three representative animals, showing some individual variation. (**B**) Transmission electron microscopy (TEM) analysis of EVs derived from reindeer’s plasma; the scale bar in all images represents 50 nm. (**C**) Western blotting analysis (WB) showing reindeer plasma EVs positive for CD63 and Flot-1; the molecular weight standard is indicated in kilodaltons (kDa). (**D**) Scatter plot representing EV number in reindeer plasma, showing some variation in plasma EV concentration between animals (*n* = 16). (**E**) Modal size of EVs in plasma of individual animals indicates some individual variation, with modal plasma EV size in the range of 110–150 nm, similar as seen in the NTA graphs in A; (*n* = 16). Error bar in D and E represents standard deviation (SD).

The NanoSight NS300 was utilized for NTA assessment of particle numbers and size distribution of reindeer plasma EVs. These were found to be poly-dispersed in the size range of 40–500 nm, with the majority of the EVs in the size range of 100–250 nm (Figure 1A). Transmission electron microscopy (TEM) confirmed EV morphology (Figure 1B) and Western blotting confirmed positive signal with two phylogenetically conserved EV-specific markers, CD63 and Flot-1 (Figure 1C). EV yield from plasma of the different individuals showed some variability within the range of 4.72 × 10^9^–3.11 × 10^10^ particles/mL (Figure 1D) and modal EV size was in the range of 110–156 nm (Figure 1E).

### 3.2. PAD Protein Homologue and Deiminated Proteins in Reindeer Plasma and Plasma EVs

**Figure 2:**
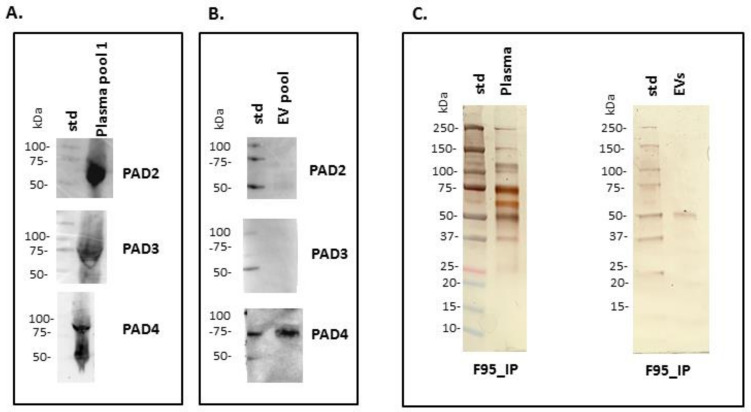
Peptidylarginine deiminase (PAD) isoforms and deiminated proteins in reindeer plasma and plasma EVs. (**A**) Positive bands identified in reindeer plasma at expected size range of approximately 70–75 kDa using anti-human PAD2-, PAD3- and PAD4-specific antibodies (a pool of five plasma is represented). (**B**) In plasma EVs, PAD2 and PAD3 isozymes were not detected while a strong positive reaction for PAD4 was observed (EV pool from five animals). (**C**) F95-enriched IP fractions, representative of deiminated protein enrichment and isolated from reindeer plasma and plasma EVs, were stained with silverstaining following SDS-PAGE in 4–20% TGX gels. The protein size standard (std) is indicated on the blots and gels in kilodaltons (kDa).

Anti-human PAD2-, PAD3- and PAD4-specific antibodies were used in Western blotting for assessment of putative PAD protein homologues in reindeer, based on cross-reaction. A positive protein band at the expected approximate 70–75 kDa size was identified for all three isozymes in plasma; in plasma EVs only PAD4 was positive, while neither PAD2 nor PAD3 where detected in plasma EVs (Figure 2A,B). To assess the presence of total deiminated proteins in plasma and plasma EVs, the F95-enriched fractions were silver stained, revealing protein bands between 25–250 kDa in plasma and 50–150 kDa in plasma EVs, respectively (Figure 2C).

**Figure 3:**
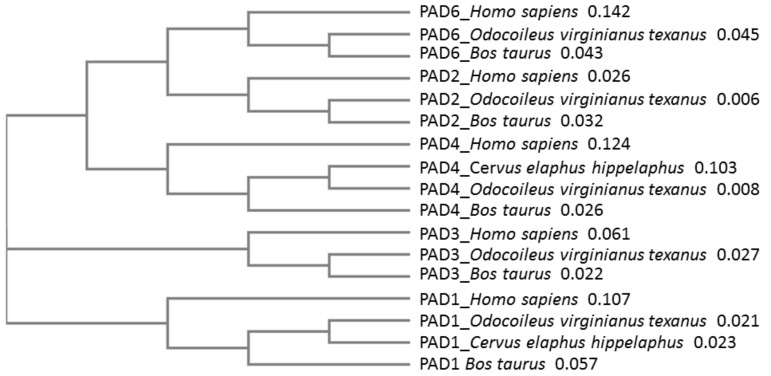
Phylogeny tree of known PADs from Artiodactyla, compared with human. White-tailed deer (*Odocoileus virginianus texanus*), red deer (*Cervus elaphus hippelaphus*) and cow (*Bos taurus*) peptidylarginine deiminase (PAD) isozymes were compared with human PADs. The following sequences were used for the neighbor joining tree construction (using Clustal Omega): *Odocoileus virginianus texanus* PAD1 (XP_020733655.1), PAD2 (XP_020733656.1), PAD3 (XP_020733658.1), PAD4 (XP_020754850.1) and PAD6 (XP_020754849.1) isozymes; *Bos taurus* PAD1 (NP_001094742.1), PAD2 (NP_001098922.1), PAD3 (XP_010800991.1), PAD4 (NP_001179102.1) and PAD6 (XP_002685843.1) isozymes; *Cervus elaphus hippelaphus* PAD1 (OWK12974.1) and PAD4 (OWK12644.1) isozymes; human (*Homo sapiens*) PAD1 (NP_037490.2), PAD2 (NP_031391.2), PAD3 (NP_057317.2), PAD4 (NP_036519.2) and PAD6 (NP_997304.3) isozymes. The numbers next to the species names represent a measure of support for the node.

Sequence alignment, followed by neighbor joining tree construction comparing known PAD isozyme sequences from deer (white-tailed deer, red deer) with cattle (*Bos taurus*) and human (*Homo sapiens*) PADs, further revealed that deer PAD isozymes align with other mammalian PADs (Figure 3). Based on percent identity matrix (using Clustal Omega 2.1), homology of PAD isozymes from deer (using the protein sequences from *Odocoileus virginianus texanus*) compared with human PADs was as follows: PAD1 78.88%, PAD2 93.29%, PAD3 87.71%, PAD4 75.97%, and PAD6 71.55%.

### 3.3. LC–MS/MS Analysis of Deiminated Proteins in Reindeer Plasma and Plasma EVs

**Figure 4:**
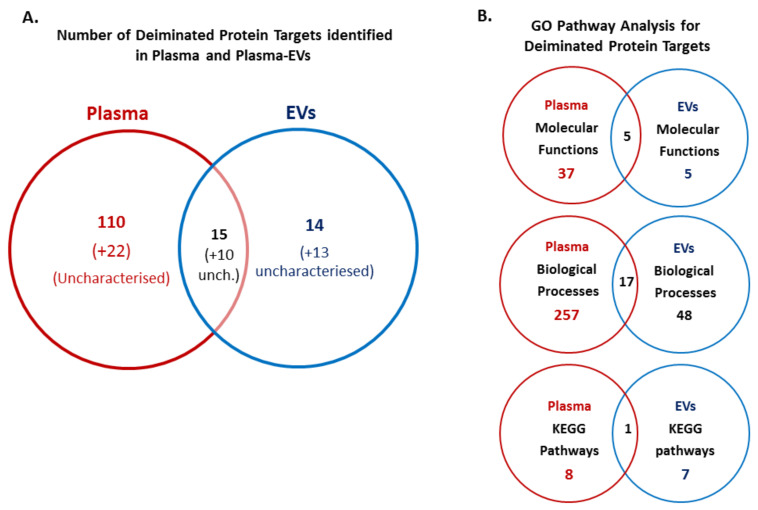
Deiminated protein hits identified in reindeer plasma and plasma EVs. (**A**) Venn diagram showing deiminated protein hits identified in *R. tarandus* whole plasma and plasma EVs, representing shared and unique proteins hits (uncharacterized hits are indicated in brackets). (**B**) Venn diagrams showing GO pathway analysis for deiminated proteins identified in plasma and plasma EVs, respectively. The number of Molecular function pathways, Biological Processes and KEGG pathways, which were found enriched in *R. tarandus* whole plasma and plasma EVs, respectively, as well as shared pathways between whole plasma and plasma EVs, are indicated.

Identification of deiminated proteins in reindeer plasma and plasma EVs was carried out following F95 enrichment using LC–MS/MS analysis. Deiminated protein hits identified in EVs, showing both hits with *R. tarandus* and other Artiodactyla, are presented in Table 1 (for full detailed LC–MS/MS data on F95-enriched proteins from plasma EVs, see Supplementary Table S1). Deiminated protein hits identified in whole plasma, showing both hits with *R. tarandus* and other Artiodactyla, are presented in Table 2 (for full detailed LC–MS/MS data on F95-enriched proteins from whole plasma, see Supplementary Table S2). The number of deiminated protein hits identified in whole plasma and plasma EVs from Table 1 and Table 2, as well as overlapping hits, are presented in the Venn diagram in Figure 4A.

Protein hits identified to be deiminated showed 15 shared identified protein hits in plasma and plasma EVs; these were albumin, serum albumin, IF rod domain, keratin, keratin 75, keratin, type I cytoskeletal 15, bradykinin, TAF domain-containing protein, histone H4, annexin, junction plakoglobin, VH region, Ig-like domain, endoplasmic reticulum chaperone and obscurin. For whole plasma, 110 hits were identified as specific, while 14 deiminated protein hits were identified to be specific to EVs only (Figure 4A; for identification of specific hits, see highlighted proteins in Table 1 and Table 2). EV-specific hits included keratins (KRT5, KRT17, KRT19), collagen (type I alpha-1 and alpha 2 chain; type III alpha-1 chain and isoform X1), SH3 domain-containing protein, cytoplasmic actin 1, endoplasmic reticulum chaperone BiP, HATPase c domain-containing protein, ubiquitin-60S ribosomal protein L40, lysozyme, and histone H2B (although a H2B-like protein did also come up as a possible secondary hit for an uncharacterized hit in whole plasma).

Upon protein network analysis for deimination-enriched proteins in whole plasma and plasma EVs, a number of shared and unique GO molecular function, GO biological processes and KEGG pathways were identified as represented by the Venn diagrams in Figure 4B.

### 3.4. Protein–Protein Interaction Network Identification of Deiminated Proteins in Reindeer Plasma and Plasma EVs

**Figure 5:**
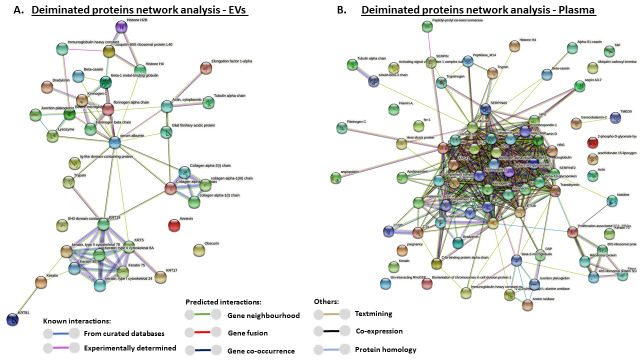
Protein–protein interaction networks of deiminated proteins identified in reindeer plasma EVs and in whole plasma. Protein–protein interaction networks for deiminated proteins in reindeer plasma EVs and whole plasma, based on known and predicted interactions in *Bos Taurus* as a representative species for Artiodactyla, using Searching Tool for the Retrieval of Interacting Genes/Proteins (STRING) analysis. PPI enrichment *p*-value for both networks is *p* < 1.0 × 10^−16^. (**A**) Protein networks enriched in deiminated proteins in reindeer plasma EVs; colored nodes represent query proteins only. (**B**) Protein networks enriched in deiminated proteins in reindeer whole plasma; colored notes represent query proteins only. Colored lines connecting the nodes show the type of interactions between the nodes in the networks; this is based on known interactions, predicted interactions and other (including textmining, co-expression and protein homology); the color code is provided in the figure.

For the prediction of protein–protein interaction networks of the deimination candidate proteins identified in plasma as well as plasma EVs, the protein names were submitted to STRING (Search Tool for the Retrieval of Interacting Genes/Proteins) analysis (https://string-db.org/). A functional protein network analysis was carried out as follows: Protein interaction networks were built based on known and predicted interactions and represent all deiminated proteins identified in *R. tarandus* plasma and plasma EVs, respectively. The interaction networks were based on proteins from the STRING protein database for *Bos taurus* as a representative species for the Phylum Artiodactyla and for a maximum number of hits, as protein identifiers for *R. tarandus* are not available in the STRING. Protein interaction networks enriched in deiminated proteins are represented below for plasma EVs and total plasma, respectively (Figure 5A,B). For both networks, the PPI enrichment *p*-value was found to be *p* < 1.0 × 10^−16^, which indicates that these proteins are biologically connected as a group and show more interactions among themselves than what would be expected for a random set of proteins of similar size, drawn from the genome.

**Figure 6:**
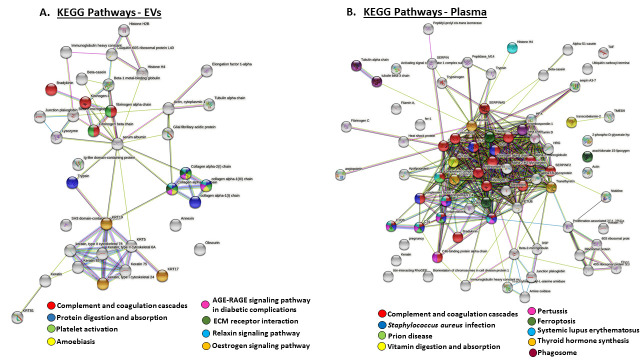
KEGG pathways for deiminated proteins identified in reindeer plasma EVs and whole plasma. Protein–protein interaction networks identified in reindeer plasma are based on known and predicted interactions in *Bos taurus* as a representative Artiodactyla for creation of the protein networks, using Searching Tool for the Retrieval of Interacting Genes/Proteins (STRING) analysis. (**A**) KEGG pathways in plasma EVs are highlighted, with eight pathways identified for the deimination protein networks. (**B**) KEGG pathways in whole plasma are highlighted; nine pathways were identified for the deimination protein networks. The color code for the specific KEGG pathways for both networks is indicated in (**A**,**B**), respectively.

STRING analysis was further used to identify KEGG pathways (Kyoto Encyclopaedia of Genes and Genomes pathways) for the deiminated protein candidates in plasma EVs and whole plasma, and these are highlighted in Figure 6. In plasma EVs, eight KEGG pathways were identified (Figure 6A), while nine KEGG pathways enriched in deiminated proteins were identified in whole plasma (Figure 6B); the only common pathway between plasma EVs and whole plasma was the complement and coagulation pathway (Figure 6). KEGG pathways identified in EVs were: protein digestion and absorption, platelet activation, amoebiasis, the AGE–RAGE signaling pathway in diabetic complications, ECM receptor interaction, the relaxin signaling pathway and the estrogen signaling pathway (Figure 6A). In whole plasma, deimination-enriched KEGG pathways (in addition to the complement and coagulation cascade) were: *Staphylococcus aureus* infection, prion diseases, vitamin digestion and absorption, pertussis, ferroptosis, SLE, thyroid hormone synthesis and phagosome (Figure 6B).

Protein networks of deiminated proteins in plasma EVs were also analyzed for GO biological processes and GO molecular function and STRING protein networks for these processes are provided in Supplementary Figure S1A,B, respectively. Similarly, whole plasma deimination STRING protein networks for GO biological processes and GO molecular function are provided in Supplementary Figure S2A,B respectively.

## 4. Discussion

This is the first study to assess extracellular vesicles (EV) and protein deimination signatures in reindeer plasma and plasma EVs. The current study aimed to provide novel insights into roles for post-translational regulation of reindeer immunity and metabolism while also highlighting putative roles for post-translational deimination in the functional diversification of conserved protein pathways throughout phylogeny.

Reindeer plasma EVs showed a poly-dispersed population in the size range of 40–500 nm with the majority of EVs falling in the range of 100–250 nm, which is a similar size distribution as previously described for *Bos taurus* plasma EVs [20], and similar as observed for human EVs. Reindeer plasma EVs showed positive for the phylogenetically conserved EV-specific markers CD63 and Flotllin-1, and were furthermore verified by transmission electron miscopy.

PAD isozymes were assessed in both reindeer plasma and plasma EVs by Western blotting, using anti-human PAD2-, PAD3- and PAD4-specific antibodies, revealing the presence of these three PAD isozymes in reindeer whole plasma at the predicted size of 70–75 kDa as seen for other mammals, while only PAD4 was shown to be exported in reindeer plasma EVs. This may be of considerable interest as the different PAD isozymes vary in their specificity for target proteins, with PAD4 having a narrower target selection than PAD2 [35], and this may therefore also reflect some of the differences observed in deiminated proteins found inside EVs, compared with deiminated proteins in whole plasma. Additionally, in comparison with previous assessment of alligator plasma EVs, where PAD2 was found in abundance in EVs, alongside low levels of PAD3 but no PAD4 export the EVs, such differences in EV-mediated PAD isozyme transport indicates differences in PAD mediated communication via EVs across animal phyla and may contribute to immune diversity observed across phylogeny. It must be noted that neither PAD1, which is associated mainly with skin and skin diseases, or PAD6, which is linked to fertility and pre-implantation embryo, were assessed in reindeer plasma in the current study.

To identify deiminated protein targets in plasma and plasma EVs of reindeer, F95 enrichment with tandem mass spectrometry was carried out. This analysis revealed some differences between hits in whole plasma and plasma EV cargo: overall, 110 deiminated proteins were identified to be specific to whole plasma and 14 deiminated proteins were identified in plasma EVs only; in addition, 15 deiminated protein hits were found to be shared between whole plasma and plasma EVs. Further uncharacterized proteins were also identified in both plasma and plasma EVs. Overall, this indicates differences of deimination mediated functions in cellular communication via EVs, compared with whole plasma, and functional protein network analysis for these deiminated protein hits in reindeer plasma and plasma EVs was therefore performed using STRING analysis. This revealed differences in deimination enrichment in functional protein networks of Kyoto Encyclopaedia of Genes and Genomes (KEGG) pathways, as well as Gene Ontology (GO) pathways for biological processes and molecular functions as discussed below: 

KEGG pathways shared for deiminated proteins in whole plasma and plasma EVs were complement and coagulation pathways. Additional KEGG pathways which were specific for deiminated proteins in plasma EVs included ECM receptor interaction, platelet activation, amoebiasis, the estrogen signaling pathway, the AGE–RAGE signaling pathway in diabetic complications, the relaxin signaling pathway, as well as in protein digestion and absorption. KEGG pathways specific for deiminated proteins in whole plasma were pertussis, ferroptosis, phagosome, *Staphylococcus aureus* infection, systemic lupus erythematosus (SLE), prion disease, thyroid hormone synthesis, vitamin digestion and absorption.

Of interest is that while the complement and coagulation system was identified as deiminated in both whole plasma and plasma EVs, differences were observed in the target proteins of deimination which participate in these cascades. In plasma EVs, proteins connected to complement and the coagulation system and identified as deimination candidates were fibrinogen, kinogenin and bradykinin. The complement system has important roles in clearing invading pathogens, as well as necrotic and apoptotic cells, and bridges innate and adaptive immunity [62]. In whole plasma, key complement components including C1q, C3, C4, C5, C9, as well as factor H and the C3/C5 convertase were identified as deiminated. Deimination of complement components has previously been reported for a various complement components, including some identified here, in serum and plasma from a range of species [12,13,14,15,16,17,18,19,20,21,22,23,24,25,29]. Interestingly, in bovine serum-EVs, a number of deiminated complement components has been identified, including C1q, C3, C4A, C5a, C7, C8, C9 factor B, Factor H, C4-binding protein [20], while in teleost fish serum both C3 and C4 were identified in deiminated form but in teleost serum-EVs C3 was more dominant in deiminated form, compared with C4 [12,13,16]. This indicates that deimination mediated regulation of complement-related mechanisms, including via EVs, may differ between animal species and may possibly also link to different export of PAD isoforms in EVs between species—also bearing in mind that lower in phylogeny, only one PAD form will be responsible for all deimination, while in mammals there are more PAD isoyzmes with target-specific preferences.

In EVs, deimination enrichment for amoebiasis and platelet activation KEGG pathways were identified, both of which have also been found deiminated in cattle [20] and relate to anti-pathogenic and injury responses. The ECM receptor interaction KEGG pathway was also identified as enriched in deiminated proteins and plays multifaceted roles (both direct and indirect) in apoptosis, cell adhesion, cell differentiation, migration and proliferation. The ECM receptor interaction pathway is also related to anti-bacterial and anti-viral responses [63,64], as well as cancer [65]; furthermore, deimination enrichment in this pathway has been identified in brain cancer [50]. The ECM receptor interaction pathway has been found enriched in deiminated proteins in cattle [20], in long lived and cancer resistant animals such as whale [14] and long-lived birds such as albatross [25], as well as in alligator, an animal with unusual anti-bacterial and anti-viral responses [18]. In EVs, the estrogen signaling pathway was identified to be enriched in deiminated proteins, and this was also previously observed in bovine plasma EVs [20]. Deimination in estrogen signaling may be of considerable interest as estrogen receptors are expressed broadly in innate and adaptive immune-related cells and also affect cytokine production, and are furthermore involved in immune regulation in the tumour environment [66]. The AGE–RAGE signaling pathway, also enriched in deiminated proteins in EVs, relates to diabetic complications [67], age- and stress-related arterial diseases [68] as well as playing multifaceted roles in cancer progression, including cell death control (apoptosis, autophagy and necroptosis), cytokine release and EMT, including in chronic mucosal inflammation [69,70,71]. Deimination enrichment in the relaxin signaling pathway was also identified to be specific to plasma EVs. This may be of considerable interest as relaxin mediates a range of biological functions including anti-apoptotic, anti-fibrotic, angiogenic, vasodilatory and anti-inflammatory responses [72]. Metabolic-related KEGG pathway identified as enriched in deiminated proteins for plasma EVs was for protein digestion and absorption. As the regulation of protein metabolism in ruminants has been studied due to importance in farming [73], putative signaling regulation in this pathway via deimination may be of some interest.

In plasma, the specific deimination-enriched KEGG pathways, besides complement and coagulation cascade, related to both immunity and metabolism. Immune-related KEGG pathways were ferroptosis, phagosome, pertussis, *Staphylococcus aureus* infection, systemic lupus erythematosus (SLE) and prion disease. This indicates a number of deimination-regulated pathways involved both in antimicrobial responses as well as autoimmunity and neurodegeneration. Cell death via ferroptosis is an iron-dependent process which occurs via iron accumulation and lipid peroxitation, resulting in oxidative cell death and related to a range of pathological processes, including nervous system disease, ischemia-reperfusion and cancer [74,75]. Therefore, insights into roles for deimination may be of considerable interest in this type of cell death. SLE is a known deimination-related autoimmune disease, alongside a number of other connective tissue autoimmune diseases [76], and therefore enrichment in autoimmune pathways was not unexpected. Similarly, prion diseases have been linked to deimination and are further discussed in detail below. Metabolic pathways enriched in deiminated proteins in plasma were thyroid hormone synthesis and vitamin digestion and absorption. The thyroid hormone synthesis pathway plays roles in the regulation of metabolism and energy homeostasis, regulation of insulin and thermogenesis, and is also linked to stress [77,78], alongside a range of pathologies including cancer, obesity, dyslipidemia, degenerative brain disease and dementia [79]. Regulation of the thyroid hormone synthesis pathway via epigenetic modifications of histones has also been reported [80] and this pathway has previously been linked to deimination enrichment in whales [14]. Furthermore, PADs and deimination have been linked to autoimmune thyroid disease and thyroid cancer [81,82]. Deimination enrichment in the vitamin digestion and absorption pathway may furthermore contribute to diverse roles of vitamin processing, which in reindeer is critical as they have undergone adaptions for vitamin D metabolism [1], and indeed vitamin D-binding protein was identified as a deimination candidate in whole plasma. Interestingly, vitamin D-binding protein has also been identified as a deimination candidate in camelids [19], which are also metabolically adapted to extreme environments.

Numerous GO biological pathways were furthermore identified in plasma and plasma EVs (Supplementary Figures S1A and S2A), with 257 pathways in plasma and 48 in EVs, while 17 were shared between plasma and plasma EVs. Shared pathways were: tricovalent inorganic cation transport, blood coagulation, cytolysis, regulation of body fluid levels, response to alcohol, iron ion homeostasis, digestion, negative regulation of developmental process, metal ion transport, iron ion transport, platelet activation, negative regulation of multicellular organismal process, metal ion homeostasis, positive regulation of multicellular organismal process, response to endogenous stimulus, response to abiotic stimulus.

Molecular function GO pathways identified for F95-enriched proteins and shared for whole plasma and plasma EVs (Supplementary Figures S1B and S2B) were: metal ion binding, identical protein binding, cysteine-type endopeptidase inhibitor activity, serine-type endopeptidase activity and structural molecule activity. In whole plasma, 42 GO molecular pathways were identified for deiminated proteins, 5 of which were shared with EVs. Molecular GO pathways specific for deiminated proteins in EVs were: the platelet-derived growth factor-binding pathway, extracellular matrix structural constituent, pyridoxal phosphate binding, protease binding and protein binding (Supplementary Figures S1 and S2).

Deimination enrichment in the various metabolic and immune-related pathways identified here may be of considerable relevance for physiological and pathobiological processes in reindeer. Importantly, reindeer have undergone a number of immune as well as metabolic adaptions, including for fat metabolism processes, limited heat loss and low resting metabolic rate, as well as changes to their internal biological clock [1,2]. Comparative genome analysis of reindeer has identified specifically adopted factors involved in immunity, vitamin D metabolism, retinal development, circadian rhythm, tolerance to cold-triggered pain and antler development [1]. Indeed, we identified deimination enrichment in numerous metabolic pathways, including vitamin and lipid metabolic pathways such as vitamin digestion and absorption, regulation of lipoprotein, lipid metabolism, cholesterol transport and efflux, fatty acid biosynthesis, regulation of ketone metabolic processes, lipid and vitamin binding. Other deimination-enriched pathways included protein digestion and absorption, the estrogen signaling pathway and thyroid hormone synthesis, as discussed above. Interestingly also, some differences were again observed between whole plasma and plasma EVs, indicating some differences in cellular communication in metabolic processes relating to EVs, as listed above (see also Supplementary Figures S1 and S2 for GO biological and GO molecular pathways).

A large number of pathways relating to defense and stress responses, innate and adaptive immunity, including the activation and regulation of immunity, humoral antimicrobial immunity, response to bacterium and parasites, complement-related functions, apoptosis and phagocytosis, iron metabolism, cytokine regulation as well as symbiosis, were here among many other immune-related pathways found to be enriched in deiminated proteins (Figure 6; Supplementary Figures S1 and S2). Furthermore, histones, which are known to act as anti-pathogenic agents. were here found to be deiminated in plasma and plasma EVs. While mainly histone H3 deimination has been studied in anti-pathogenic responses relating to extracellular trap formation (NETosis/ETosis), roles for antimicrobial effects of H2 and H4 histones has also been established, including anti-viral ones [83]. Their deimination in anti-pathogenic responses remains to be further investigated, but has been linked to gene regulatory events, including in cancer [84]. In plasma, a number of serpins were identified to be deiminated. These serine proteinases have multifaceted roles in protease inhibition, chromatin organization, hormone transport, control of apoptosis, as well as in anti-microbial and anti-viral responses [85,86,87,88]. Overall, our findings point to roles for deimination in immune response modulation, possibly allowing for protein moonlighting in health and disease and in response to various pathogenic infections via this post-translational modification. Indeed, reindeer have been widely studied in relation to a number of naturally occurring infections including parasitic bacterial and viral ones and are also related to a range of zoonotic diseases [8]; these are summarized below:

With respect to parasitic infections, more than 100 parasite species have been reported to infect or infest reindeer [89,90,91,92,93,94], many of which are also shared with other ruminants. Protozoan parasites include *Eimeria* species [95], *Cryptosporidium* and *Giardia* [96], *Entamoeba* [97], *Besnoitia* [98,99] and *Toxoplasma gondii* [100,101,102,103]. Reindeer in Fennoscandia are intermediate hosts for *Sarcocystis* spp. [104,105]. Haematozoan parasites include *Babesia* spp. and *Trypanosoma* spp. [94]. The rumen fluke *Paramphistomum leydeni* and the liver fluke *Dicrocoelium dendriticum* (Trematoda) also infect reindeer [106,107]. Furthermore, a number of Cestoda paraitize reindeer, acting either as intermediate or final hosts [91,108,109,110]. Nematoda form the most common and versatile group of parasites in reindeer and are most often associated with intestine [91,93,107,111,112,113,114,115], but can also be found in the capillaries of the ears and eyelids [116,117], or be bloodborne [118]. Some nematodes are also confined to the central nervous system (CNS) and can cause paralysis; a major concern in reindeer breeding [119], while others affect the lung [91,120,121]. Additionally, the arthropod sinus worm *Linguatula arctica* is widespread and common parasite of reindeer [91,122] and ectoparasites also infest reindeer [67], causing skin and mucosal swelling, bleeding and affect breathing [91,123,124,125]. Importantly, parasitic infections from reindeer can also be zoonotic, for example *Enterocytozoon bieneusi*—a microsporidia and obligate parasite infecting intestinal cells, is suggested to be transmitted to humans [126].

A number of naturally occurring viral infections have also been identified in reindeer, including alphaherpesvirus, bluetongue virus, malignant catarrhal fever (MCFV-)-related gammaherpesvirus, pestivirus, Schmallenberg virus [7,8,127,128,129], West Nile virus which leads to lymphohistiocytic encephalomyelitis [7] and tick-borne encephalitis virus (TBEV), which belongs to the most important neurological pathogens transmitted by tick bites in Europe [130]. Further viruses include papillomaviruses, parvovirus, and polyomavirus, as well as importantly also Coronaviridae [131]. Experimental viral infections in reindeer include herpesvirus 2 and parapoxvirus, both of which have though also been detected in Norwegian reindeer [129], including semi-domesticated reindeer [132] as well as in Alaskan caribou and other wildlife, and are known to be transmitted between wildlife, sheep, goats and human [5]. 

Importantly, the white-tailed deer (*Odocoileus virginianus*) has recently been proven to be experimentally infected via intranasal inoculation with SARS-CoV-2, showing evidence of subclinical viral infection as well as shedding of infectious virus in nasal secretions and feces, as well as detection of viral RNA in multiple tissues [9]. These findings point to deer as a putative new zoonotic host for the virus, although it still needs to be further established whether the infection will also happen naturally, and therefore contribute as a novel viral reservoir [9]. Whether there is a possibility of zoonotic transmission from deer back to humans, as recently observed in mink [133,134,135], also needs to be established, as well as if other Cervidae, including reindeer, can act as zoonotic hosts and reservoirs for SARS-CoV-2. Furthermore, it must be considered that reindeer do come in close contact with sheep through shared grounds for grazing, and may also encounter other domestic or wild animals on shared habitats elsewhere, and this could possibly be another concern regarding zoonosis spread.

Bacterial infections described in reindeer include *Anaplasma phagocytophilum*, which in sheep and cattle causes tick-borne fever and can in human cause the zoonotic disease granulocytic anaplasmosis [8]. Evidence for transmission of Lyme disease, which is caused by *Borrelia bugdorferi*, a tick-borne encephalitis associated bacterium, has furthermore been described from reindeer to human following skinning of a reindeer, causing meningoencephalitis [6]. Reindeer are also subject to tuberculosis, caused by *Mycobacterium bovis* [136], as well as polymicrobial bronchopneumonia caused by *Mycoplasma ovipneumoniae* [137]. *Brucella* spp., is a zoonotic bacteria that is one of the most widespread and economically impactful zoonosis affecting reindeer and can be transmitted to human via raw animal products, including from reindeer and caribou [138,139]. Anthrax, a global zoonotic and epizoonotic disease, is another bacterial pathogen identified in reindeer, particularly in relation to infected carcasses, as recently found in Siberian permafrost and therefore also indicative of a possible rise in the Arctic due to climate change [140]. Reindeer have in addition been found to carry *Clostridium perfringens* [141] and can suffer from severe bacterial intestinal infections and endotoxemia by *Clostridium* sp [142,143], while other bacteria inside the order Clostridiales aid in processing of lichen secondary metabolites [144]. Reindeer also carry *Erysipelothrix rhusiopathiae-* which relates to urticaria-like lesions, arthralgia, arthritis, endocarditis and sepsis and can furthermore be transmitted to humans [145]. This may be of interest as both bacterial and autoimmune disease pathways were here identified to be linked to deiminated proteins in reindeer plasma via STRING analysis.

The strong relationship of deimination-enriched proteins with a number of immune-related pathways may therefore be important in relation to the infections listed above, both naturally occurring ones as well as zoonotic ones. It also has to be considered that PADs are phylogenetically conserved proteins which are also found in bacteria and parasites, both of which can use their PAD homologues to manipulate host immunity [39,40]. Therefore the interplay of host–pathogen post-translational regulation is a field which requires further investigation.

Importantly, in the current study, deimination-enriched pathways were also identified for prion disease and amyloid in whole plasma. This is indicative of that deimination plays roles in prion diseases in reindeer. Deer are well known to be affected by prion-related transmissible spongiform encephalopathy, a neurodegenerative disease which in reindeer manifests as chronic wasting disease (CWD), and is also found in deer, elk and moose [4,146,147]. Its transmission is believed to be primarily transmitted via direct contact of oral and mucosal membranes between positive and susceptible animals, also via feces and urine, as well as prion reservoirs in soil and water [148,149]. In white tailed deer, differential gene expression analysis for CWD has identified links to various cellular components, as well as retroviral infection [150]. Previous studies have indeed related protein deimination to prion disease including Creutzfeldt-Jacob Disease and scrapie [151,152,153,154,155], via effects on prion conformation, enolase, protein accumulation and pathogenesis, although further in depth examination into exact mechanistic pathways is still needed. Furthermore, a recent animal study on early pre-motor Parkinson’s disease (PD) identified enrichment of deiminated proteins in pathways relating to prion disease in the PD, versus control animals [156], further highlighting deimination as a common factor in various neurodegenerative disorders, including prion diseases. It may therefore be of considerable importance that deimination was identified here also in prion disease-related pathways in reindeer as this shows conserved pathways across phyla. Furthermore, the disease is spreading geographically including in the US, Canada, Scandinavia and South Korea [147] and therefore understanding underlying pathways to develop measures for reducing transmission between animals, including domestic ones such as cattle, sheep and swine, as well as possible transmission to human, including via consumption of infected deer and elk, is of considerable importance [149,157,158,159,160].

It must be noted that the analysis of protein networks enriched in deiminated proteins presented here relied on deimination enrichment using the pan-citrulline-specific F95 antibody, and therefore further evaluation, including that of individual candidate proteins for deimination and using other citrulline signature proteomic approaches, should also be considered. The protein networks presented are based on functional protein network analysis, which besides experimentally established interactions also takes into account indirect and functional interactions. The protein network analysis is furthermore based on protein hits identified in the Artiodactyla UniProt database, and using *Bos taurus* as a representative species for Artiodactyla, as a species-specific reindeer proteomic database is not available in STRING. It must also be considered that the current study only assessed female reindeer that were assessed as healthy, and therefore further investigations into deimination and EV signatures relating to sex differences and health status remain subject to further in depth investigations. Nonetheless, this study provides a base line for furthering understanding of the roles of deimination and EVs in reindeer immunity and metabolism and may provide a platform for the development of novel biomarkers to assess wild life health status and zoonotic disease transmission.

## 5. Conclusions

The current study characterized PAD expression and deiminated protein product signatures in plasma and plasma extracellular vesicles (EVs) of reindeer (*R. tarandus*). Some differences were observed for PAD isozyme PAD2, PAD3 and PAD4 detection in whole plasma versus plasma EVs, with only PAD4 being exported in the EVs. Protein deimination signatures of whole plasma versus plasma EVs confirmed a range of KEGG and GO pathways relating to key immune and metabolic functions, including pathways for innate and adaptive immunity, prion disease, as well as hormonal regulation, vitamin and lipid metabolism. This provides novel insights into the roles for post-translational protein deimination in the regulation of key pathways involved in physiological and pathophysiological processes and further links to various known pathways in deer relating to infection and immunity in pathogenic and prion diseases. Furthermore, deimination may contribute to various unique adaptions of reindeer immune and metabolic pathways, which warrants further investigation. Importantly, as reindeer can play roles as reservoir hosts for a range of pathogens circulating in captive, wild as well as domestic ruminant species, and be a reservoir for zoonotic disease, including coronaviruses, bacterial and parasitic ones, the current study provides novel insights into their immune systems, which may be critically relevant in understanding of zoonosis spread and management.

## Figures and Tables

**Table 1:** Deiminated proteins in plasma EVs of reindeer (*R. tarandus*), identified by F95 enrichment and liquid chromatography with tandem mass spectrometry (LC–MS/MS) analysis. Proteins identified only in plasma EVs (and not in whole plasma) are highlighted in blue and with an asterix (*); uncharacterized hits with a secondary hit that was annotated are included and indicated in brackets. Protein ID, protein name, species hit with the Artiodactyla UniProt database, number of matches and total score are included in the table. For full detailed LC–MS/MS data for F95-enriched proteins, see Supplementary Table S1.

Protein ID Protein Name	*Species Name* Common Name	Matches (Sequences)	Total Score (*p* < 0.05) ^†^
**A0A140T897_BOVIN** *Albumin*	*Bos taurus* Cow	2 (39)	2322
**L8ISP4_9CETA** *Serum albumin*	Bos mutus Domestic Yak	107 (37)	2198
**A0A4W2GW83_BOBOX** *Uncharacterized protein (ALB protein)*	*Bos indicus x Bos taurus* Zebu x Cow	105 (37)	2072
**A0A5N3XZ04_MUNRE** *IF rod domain-containing protein*	*Muntiacus reevesi* Chinese muntjac	44 (25)	1128
*A0A212DF80_CEREH *KRT*5	*Cervus elaphus hippelaphus* European red deer	44 (25)	1275
**A0A6J0WT46_ODOVR** *Serum albumin*	*Odocoileus virginianus texanus* White-tailed deer	52 (22)	1177
**A0A5N4DHW9_CAMDR** *Keratin*	*Camelus dromedarius* Dromedary	40 (21)	1116
**A0A4W2C021_BOBOX** *Uncharacterized protein (collagen alpha-1* *(I) chain)*	*Bos indicus x Bos taurus* Zebu x Cow	34 (20)	1080
**A0A452FHU9_CAPHI** *Uncharacterized protein (collagen type I alpha 1 chain)*	*Capra hircus* Goat	34 (16)	988
**A0A5N3WTF4_MUNMU** *Uncharacterized protein (collagen alpha-1(I) chain isoform X1)*	*Muntiacus muntjac* Barking deer	23 (13)	976
**A0A5N4D320_CAMDR** *Keratin*	*Camelus dromedarius* Dromedary	37 (15)	832
**A0A6J0WBI9_ODOVR** *Histidine-rich glycoprotein isoform X1*	*Odocoileus virginianus texanus* White-tailed deer	33 (14)	806
*A0A212D793_CEREH *KRT19*	*Cervus elaphus hippelaphus* European red deer	32 (13)	785
**A0A287B5W2_PIG** *Trypsinogen isoform X1*	*Sus scrofa* Wild boar	185 (157)	765
**A0A4W2D3K5_BOBOX** *Keratin 75*	*Bos indicus x Bos taurus* Zebu x Cow	33 (13)	729
**A0A4W2DIS9_BOBOX** *Keratin 75*	*Bos indicus x Bos taurus* Zebu x Cow	31 (15)	724
**A0A6B0R6W5_9CETA** *Uncharacterized protein (IF rod domain-containing; glial fibrillary acidic protein*)	*Bos mutus* Domestic yak	25 (14)	704
**9XAP9_CAMFR** *Keratin, type I cytoskeletal 14-like protein*	Camelus ferus Wild Bactrian camel	24 (10)	694
**A0A452FN18_CAPHI** *IF rod domain-containing protein*	*Capra hircus* Goat	13 (9)	676
**A0A5N4DGN6_CAMDR** *Keratin*	*Camelus dromedarius* Dromedary	29 (14)	670
**A0A4W2IN22_BOBOX** *IF rod domain-containing protein*	*Bos indicus x Bos taurus* Zebu x Cow	25 (11)	670
**A0A6I9IRH0_VICPA** keratin, type I cytoskeletal	*Vicugna pacos* Alpaca	10 (8)	667
**A0A5G2QXD3_PIG** *IF rod domain-containing protein*	*Sus scrofa* Wild boar	27 (18)	655
*A0A3Q1LZN8_BOVIN *Collagen alpha-2(I) chain*	*Bos taurus* Cow	26 (13)	634
**A0A287BLD2_PIG** *Uncharacterized protein (collagen alpha-1(I) chain preproprotein; alpha 1 chain of type I collagen)*	*Sus scrofa* Wild boar	19 (9)	628
*A0A212D6S5_CEREH *KRT17*	*Cervus elaphus hippelaphus* European red deer	16 (11)	580
**A0A5N4DFY6_CAMDR** *Keratin*	*Camelus dromedarius* Dromedary	19 (12)	550
**A0A212CMY9_CEREH** *Uncharacterized protein (immunoglobulin heavy constant; beta-2-microglobulin)*	*Cervus elaphus hippelaphus* European red deer	10 (8)	539
*A0A6J3QLJ4_TURTR *Collagen alpha-1(I) chain*	*Tursiops truncates* Common bottlenose dolphin	9 (7)	488
**A0A2Y9SJP9_PHYMC** *Keratin, type II cytoskeletal 6A*	*Physeter macrocephalus* Sperm Whale	26 (10)	469
**A0A452EP10_CAPHI** *IF rod domain-containing protein*	*Capra hircus* Goat	9 (7)	469
**A0A6B0R542_9CETA** *Uncharacterized protein* *(bradykinin; kininogen-1; kininogen-2)*	*Bos mutus* Wild yak	16 (8)	466
**A0A5N4DG47_CAMDR** *Keratin*	*Camelus dromedarius* Dromedary	19 (9)	463
**A0A5N3WDS4_MUNMU** *Bradykinin*	*Muntiacus muntjac* Barking deer	10 (9)	443
*A0A1S7J1Y9_PIG *Alpha2 chain of type I collagen*	*Sus scrofa* Wild boar	17 (10)	442
**A0A5N4DFY1_CAMDR** *Keratin*	*Camelus dromedarius* Dromedary	13 (8)	380
**A0A383ZWF6_BALAS** *Keratin, type II cytoskeletal 6A-like isoform X2*	Balaenoptera acutorostrata scammoni Minke whale	13 (8)	360
**W5Q4S0_SHEEP** *Uncharacterized protein (collagen alpha-1(III) chain; collagen type III alpha 1 chain; fibrillar collagen NC1 domain-containing protein)*	*Ovis aries* Sheep	7 (5)	340
**A0A2F0AVL6_ESCRO** *Keratin, type II cytoskeletal 4*	*Eschrichtius robustus* Gray whale	12 (7)	339
*A0A5N3W3N9_MUNRE *SH3 domain-containing protein*	*Muntiacus reevesi* Chinese muntjac	8 (8)	296
**A0A340XVM8_LIPVE** *Keratin, type I cytoskeletal 15*	*Lipotes vexillifer* Baiji	10 (6)	294
**A0A5N4CT25_CAMDR** *Histone H4*	*Camelus dromedarius* Dromedary	5 (5)	240
**A0A5N3XAC4_MUNRE** *Uncharacterized protein (Ig-like domain-containing protein)*	*Muntiacus reevesi* Chinese muntjac	7 (3)	239
**A0A6J0ZDI0_ODOVR** *Serotransferrin*	*Odocoileus virginianus texanus* White-tailed deer	5 (5)	234
*ACTB_BOSMU *Actin, cytoplasmic 1*	*Bos mutus grunniens* Wild yak	6 (5)	232
**A0A5N3WEA4_MUNMU** *Beta-1 metal-binding globulin*	*Muntiacus muntjac* Barking deer	5 (5)	232
**A0A6J0XRB4_ODOVR** *Keratin, type II cytoskeletal 2 oral-like*	*Odocoileus virginianus texanus* White-tailed deer	16 (6)	228
**A0A2C9F3E9_PIG** *Junction plakoglobin*	*Sus scrofa*	6 (1)	228
**A0A212DB90_CEREH** *Ig-like domain-containing protein*	*Cervus elaphus hippelaphus* European red deer	6 (3)	225
*0A6I9IE32_VICPA *Collagen alpha-1(III) chain isoform X1*	*Vicugna pacos* Alpaca	6 (4)	224
**A0A212D5P4_CEREH** *TAF domain-containing protein*	*Cervus elaphus hippelaphus* European red deer	5 (5)	222
**A0A643C4S8_BALPH** *Uncharacterized protein (IF rod domain-containing protein; KRT81; Keratin 85)*	*Balaenoptera physalus* Fin Whale	10 (6)	215
**A0A5N3W8P2_MUNMU** *Uncharacterized protein (Ig-like domain-containing protein*)	*Muntiacus muntjac* Reeves’s muntjac	7 (3)	212
**A0A212D7J2_CEREH** *Fibrinogen beta chain*	*Cervus elaphus hippelaphus* European red deer	3 (3)	192
**A0A287B7K6_PIG** *IF rod domain-containing protein*	*Sus scrofa* Wild boar	9 (6)	186
**A0A212DFA6_CEREH** *IF rod domain-containing protein*	*Cervus elaphus hippelaphus* European red deer	11 (5)	184
**A0A6J0XD83_ODOVR** *Fibrinogen alpha chain*	*Odocoileus virginianus texanus* White-tailed deer	5 (4)	180
*A0A2Y9MPQ9_DELLE *Collagen alpha-1(III) chain*	*Delphinapterus leucas* Beluga whale	5 (4)	163
**A0A452E8D3_CAPHI** *Ig-like domain-containing protein*	*Capra hircus* Goat	3 (2)	149
**W5P2K5_SHEEP** *IF rod domain-containing protein*	*Ovis aries* Sheep	7 (4)	147
**A0A5N3UHT3_MUNRE** *Ig-like domain-containing protein*	*Muntiacus reevesi* Chinese muntjac	2 (2)	147
**A2P2I1_SHEEP** *VH region*	*Ovis aries* Sheep	1 (1)	131
*Q0VCX2|BIP_BOVIN *Endoplasmic reticulum chaperone BiP*	*Bos taurus* Cow	2 (2)	101
**A0A212CAL2_CEREH** *Elongation factor 1-alpha*	*Cervus elaphus hippelaphus* European red deer	2 (2)	100
**A0A5N3UV43_MUNMU** *IF rod domain-containing protein*	*Muntiacus muntjac* Barking deer	5 (2)	89
**A0A3Q1LUE9_BOVIN** *Ig-like domain-containing protein*	*Bos taurus* Cow	1 (1)	87
**A0A6B9SDT6_BOVIN** *Ig lamda chain variable region*	*Bos taurus* Cow	1 (1)	87
**A0A212CSZ9_CEREH** *Ig-like domain-containing protein*	*Cervus elaphus hippelaphus* European red deer	2 (1)	83
**A0A2F0B9E6_ESCRO** *Trypsin*	*Eschrichtius robustus* Gray whale	2 (1)	80
*A0A286ZKC5_PIG *HATPase_c domain-containing protein*	*Sus scrofa* Wild boar	2 (0)	76
**A0A1L6BP13_BUBBU** *Beta-casein*	*Bubalus bubalis* Water buffalo	3 (2)	75
**A0A6B0S2F2_9CETA** *Fibrinogen C-terminal domain-containing protein*	*Bos mutus* Wild yak	3 (2)	68
*P0C276|RL40_SHEEP *Ubiquitin-60S ribosomal protein L40*	*Ovis aries* Sheep	1 (1)	67
*A0A2Y9SBW8_PHYMC *Histone H2B*	*Physeter macrocephalus* Sperm Whale	2 (2)	65
**A0A6B0RTH8_9CETA** *Uncharacterized protein (obscurin)*	*Bos mutus* Wild yak	2 (2)	63
**A0A6J3S691_TURTR** *Keratin, type II cytoskeletal 78*	*Tursiops truncates* Common bottlenose dolphin	2 (2)	63
**A0A383ZRF2_BALAS** *Keratin, type I cytoskeletal 24*	*Balaenoptera acutorostrata scammony* Minke whale	1 (1)	62
*A0A0C5AGQ3_BUBBU *Lysozyme*	*Bubalus bubalis* Water buffalo	1 (1)	61
**A2P2I3_SHEEP** *VH region*	*Ovis aries* Sheep	1 (1)	60
**A0A075B7I6_PIG** *Ig-like domain-containing protein*	*Sus scrofa* Wild boar	1 (1)	59
**A0A0R4I993_SUSBA** *Tubulin alpha chain*	*Sus barbatus* Bornean bearded pig	1 (1)	53
**A0A5N4EAI9_CAMDR** *Annexin*	*Camelus dromedarius* Dromedary	2 (2)	50
**A0A2Y9EH04_PHYMC** *Fer-1-like protein 4*	*Physeter macrocephalus* Sperm Whale	2 (2)	50
**A0A5J5N0U1_MUNRE** *Uncharacterized protein* (small proline-rich protein 2I-like; *Type II small proline-rich protein)*	*Muntiacus reevesi* Chinese muntjac	1 (1)	50
**A0A6B0R269_9CETA** *Ig-like domain-containing protein*	*Bos mutus* Wild yak	2 (1)	49
**A0A452E907_CAPHI** *Uncharacterized protein (skin-specific protein 32; Chromosome 3 C1orf68 homolog; Chromosome 1 open reading frame 68)*	*Capra hircus* Goat	1 (1)	48
**A0A4W2E476_BOBOX** *Ig-like domain-containing protein*	*Bos indicus x Bos taurus* Zebu x Cow	1 (1)	48
**A0A212CS30_CEREH** *Ig-like domain-containing protein*	*Cervus elaphus hippelaphus* European red deer	2 (1)	47

† Ions score is −10*Log(P), where P is the probability that the observed match is a random event. Individual ions scores > 46 indicate identity or extensive homology (*p* < 0.05). Protein scores are derived from ions scores as a non-probabilistic basis for ranking protein hits.

**Table 2:** Deiminated proteins in whole plasma of reindeer (*Rangifer tarandus*) identified by F95 enrichment and liquid chromatography with tandem mass spectrometry (LC–MS/MS) analysis. Proteins identified only in whole plasma (and not in plasma EVs) are highlighted in pink and with an asterix (*); uncharacterized hits with a secondary hit that was annotated are included and indicated in brackets. Protein ID, protein name, species hit with the Artiodactyla UniProt database, number of matches and total score are included in the table. For full detailed LC–MS/MS data on F95-enriched proteins, see Supplementary Table S2.

Protein ID *Protein Name*	*Species Name* Common Name	Matches (Sequences)	Total Score (*p* < 0.05) ^†^
*A0A6J0ZEI2_ODOVR *Complement C3*	*Odocoileusvirginianus texanus* White-tailed deer	71 (51)	3535
*A0A6J0Y2W1_ODOV *Fibronectin isoform X5*	*Odocoileus virginianus texanus* White-tailed deer	70 (50)	3454
*A0A5N3WRA9_MUNMU *C3-beta-c*	*Muntiacus muntjac* Barking deer	70 (51)	3438
*A0A6J0YF65_ODOVR *alpha-2-macroglobulin*	*Odocoileus virginianus texanus* White-tailed deer	43 (37)	2929
**A0A140T897_BOVIN** *Albumin*	*Bos taurus* Cow	81 (59)	2831
**A0A5J5N929_MUNRE** *Uncharacterized protein* *(alpha-2-macroglobulin-like)*	*Muntiacus reevesi* Chinese muntjac	59 (42)	2815
**L8ISP4_9CETA** *Serum albumin*	*Bos mutus* Domestic Yak	79 (58)	2691
*A0A6J0YGQ5_ODOVR *Pregnancy zone protein-like isoform X1*	*Odocoileus virginianus texanus* White-tailed deer	49 (38)	2596
**A0A6J0WT46_ODOVR** *Serum albumin*	*Odocoileus virginianus texanus* White-tailed deer	99 (66)	2581
**A0A6J0ZDI0_ODOVR** *Serotransferrin*	*Odocoileus virginianus texanus* White-tailed deer	77 (49)	2452
**A0A5N3XN56_MUNRE** *Beta-1 metal-binding globulin*	*Muntiacus reevesi* Chinese muntjac	65 (44)	2268
**A0A212D5P0_CEREH** *ALB*	*Cervus elaphus hippelaphus* European red deer	84 (56)	2237
**X2GM95_CERNI** *Serum albumin*	*Cervus nippon* Sika deer	82 (54)	2182
**A0A6J0XGG0_ODOVR** *Fibrinogen beta chain*	*Odocoileus virginianus texanus* White-tailed deer	63 (47)	1890
**A0A6J0XD83_ODOVR** *Fibrinogen alpha chain*	*Odocoileus virginianus texanus* White-tailed deer	62 (42)	1770
**A0A5N3WDS4_MUNMU** *Bradykinin*	*Muntiacus muntjac*	76 (49)	1754
*A0A6J0WDQ8_ODOVR *Kininogen-1 isoform X1*	*Odocoileus virginianus texanus*	72 (46)	1645
*A0A6J0WBI9_ODOVR *Histidine-rich glycoprotein isoform X1*	*Odocoileus virginianus texanus*	152 (95)	1514
**A0A5N3WD93_MUNMU** *Fibrinogen C-terminal domain-containing protein*	*Muntiacus muntjac* Barking deer	54 (43)	1470
*A0A212CD20_CEREH *2M*	*Cervus elaphus hippelaphus* European red deer	32 (24)	1469
**A0A5N3XZ04_MUNRE** *IF rod domain-containing protein*	*Muntiacus reevesi* Chinese muntjac	37 (25)	1466
**A0A5N3WGH1_MUNMU** *Uncharacterized protein (HRG* *)*	*Muntiacus muntjac* Barking deer	142 (87)	1393
**A0A6B0S2F2_9CETA** *Fibrinogen C-terminal domain-containing protein*	*Bos mutus* Wild yak	43 (29)	1365
**A0A5N4DHW9_CAMDR** *Keratin*	*Camelus dromedarius* Dromedary	31 (21)	1284
*A0A212D8V0_CEREH *FGG*	*Cervus elaphus hippelaphus*	47 (34)	1235
*A0A6J0YZJ7_ODOVR *Ceruloplasmin isoform X2*	*Odocoileus virginianus texanus* European red deer	28 (16)	1227
**A0A5N3WB21_MUNMU** *Fibrinogen alpha chain*	*Muntiacus muntjac* Barking deer	42 (24)	1207
**W5PF65_SHEEP** *Beta-1 metal-binding globulin*	*Ovis aries* Sheep	32 (21)	1206
**A0A212CMY9_CEREH** *Uncharacterized protein (immunoglobulin heavy constant mu; beta-2-microglobulin* *)*	*Cervus elaphus hippelaphus* European red deer	47 (30)	1156
**A0A5N3XTY4_MUNRE** *Uncharacterized protein* *(c* *omplement factor H* *)*	*Muntiacus reevesi* Chinese muntjac	26 (21)	1151
*A0A6J0XY06_ODOVR *Thrombospondin-1 isoform*	*Odocoileus virginianus texanus* White-tailed deer	21 (12)	1076
*A0A6J0XUD5_ODOVR *Complement C4-A-like*	*Odocoileus virginianus texanus* White-tailed deer	20 (15)	1074
*A0A6J0WY92_ODOVR *Complement factor H-like*	*Odocoileus virginianus texanus* White-tailed deer	22 (17)	1026
*A0A6J0W0N0_ODOVR *Inter-alpha-trypsin inhibitor heavy chain H1*	*Odocoileus virginianus texanus* White-tailed deer	18 (14)	944
*A0A4W2C0F6_BOBOX *C4a anaphylatoxin*	*Bos indicus x Bos taurus* Zebu x Cow	16 (11)	943
*A0A212CJ19_CEREH *CP*	*Cervus elaphus hippelaphus* European red deer	22 (13)	935
*A0A6J0XUP5_ODOVR *Complement C4-A-like*	*Odocoileus virginianus texanus* White-tailed deer	15 (12)	923
*E1BH06_BOVIN *C4a anaphylatoxin*	*Bos taurus* Cow	16 (11)	921
*A0A6J0WIC5_ODOVR *Inter-alpha-trypsin inhibitor heavy chain H2*	*Odocoileus virginianus texanus* White-tailed deer	20 (14)	879
*A0A6J0YC26_ODOVR *Heparin cofactor 2*	*Odocoileus virginianus texanus* White-tailed deer	17 (11)	830
**A0A6I9IRH0_VICPA** *Keratin, type I cytoskeletal 14*	*Vicugna pacos* Alpaca	23 (14)	822
**A0A287B5W2_PIG** *Trypsinogen isoform X1*	*Sus scrofa* Wild boar	145 (124)	795
*A0A6J0ZDS1_ODOVR *C4b-binding protein alpha chain*	*Odocoileus virginianus texanus* White-tailed deer	15 (9)	783
**A0A6J0VYI5_ODOVR** *Uncharacterized protein (complement C1q)*	*Odocoileus virginianus texanus* White-tailed deer	19 (14)	774
**A0A341C5T8_NEOAA** *Serum albumin*	*Neophocaena asiaeorientalis asiaeorientalis* Narrow-ridged finless porpoise	31 (11)	734
**A0A6J0YVR0_ODOVR** *Keratin, type I cytoskeletal 15 isoform X1*	*Odocoileus virginianus texanus* White-tailed deer	20 (13)	720
**A0A4W2D3K5_BOBOX** *Keratin 75*	*Bos indicus x Bos taurus* Zebu x Cow	22 (14)	716
**A0A287AEL2_PIG** *IF rod domain-containing protein*	*Sus scrofa* Wild boar	22 (12)	708
*A0A5N3XTJ5_MUNRE *Antithrombin-III*	*Muntiacus reevesi* Chinese muntjac	14 (4)	614
*A0A220IGA4_RANTA *Adult beta-globin*	*Rangifer tarandus* Reindeer	12 (11)	603
**A0A212CMB3_CEREH** *Uncharacterized protein* *(* *Ig gamma-3 chain C region; IgG heavy chain)*	*Cervus elaphus hippelaphus* European red deer	20 (15)	593
*A0A6J0WIA8_ODOVR *Prothrombin*	*Odocoileus virginianus texanus* White-tailed deer	12 (8)	567
**A0A6I9I3P0_VICPA** *Keratin, type II cytoskeletal 5-like*	*Vicugna pacos* Alpaca	16 (9)	563
*A0A6J0Y9J4_ODOVR *Apolipoprotein A-I*	*Odocoileus virginianus texanus* White-tailed deer	10 (7)	543
*Q9TS85_BOVIN *Histidine-rich GLYCOPROTEIN=FACTOR XIIIA substrate*	*Bos taurus* Cow	39 (18)	536
*A0A212DHP9_CEREH *APOA1*	*Cervus elaphus hippelaphus* European red deer	9 (7)	504
*A0A0B8RTA2_PIG *Actin, gamma 1*	*Sus scrofa* Wild boar	10 (6)	502
*A0A212D467_CEREH *C1QB*	*Cervus elaphus hippelaphus* European red deer	13 (9)	498
**A0A2Y9SJP9_PHYMC** *Keratin, type II cytoskeletal*	*Physeter macrocephalus* Sperm whale	14 (7)	495
*A0A5N3VLU1_MUNMU *Prothrombin*	*Muntiacus muntjac* Barking deer	11 (6)	482
**HRG_BOVIN** *Histidine-rich glycoprotein*	*Bos taurus* Cow	21 (14)	480
*S9Y253_CAMFR *Kininogen-2 isoform I*	*Camelus ferus* Wild Bactrian camel	28 (11)	451
*A0A6J0XQV8_ODOVR *Hemopexin*	*Odocoileus virginianus texanus* White-tailed deer	9 (5)	450
*A0A6J0WWF4_ODOVR *Vitronectin isoform X1*	*Odocoileus virginianus texanus* White-tailed deer	10 (7)	438
*A0A6J0W8S2_ODOVR *Plasminogen isoform X1*	*Odocoileus virginianus texanus* White-tailed deer	11 (3)	434
**A0A5J5MM15_MUNRE** *Uncharacterized protein* *(immunoglobulin kappa light chain-like)*	*Muntiacus reevesi* Chinese muntjac	12 (7)	425
*A0A6J0Z5Q2_ODOVR *Transcobalamin-2*	*Odocoileus virginianus texanus* White-tailed deer	8 (6)	423
*A0A5N3X8Z5_MUNRE *Hemopexin*	*Muntiacus reevesi* Reeves’s muntjac	7 (5)	422
*A0A212CJF4_CEREH *C1q domain-containing protein*	*Cervus elaphus hippelaphus* European red deer	17 (10)	415
*C0LXP2_ODOVR *Complement 1 subcomponent q polypeptide gamma*	*Odocoileus virginianus texanus* White-tailed deer	6 (5)	408
**A0A5N3W8P2_MUNMU** *Uncharacterized protein* *(Ig-like domain-containing protein)*	*Muntiacus muntjac* Barking deer	11 (9)	398
*A0A286ZIC1_PIG *Actin-depolymerizing factor*	*Sus scrofa* Wils boar	8 (3)	371
*A0A5N3VK90_MUNMU *Actin-depolymerizing factor*	*Muntiacus muntjac* Barking deer	7 (3)	369
**A0A212DB90_CEREH** *Ig-like domain-containing protein*	*Cervus elaphus hippelaphus* European red deer	9 (5)	364
*A0A212DHZ3_CEREH *HPX*	*Cervus elaphus hippelaphus* European red deer	7 (4)	349
*A0A6J0X6J4_ODOVR *Selenoprotein P*	*Odocoileus virginianus texanus* White-tailed deer	9 (5)	334
*A0A6J0Y2T5_ODOVR *Hemoglobin subunit alpha*	*Odocoileus virginianus texanus* White-tailed deer	7 (3)	333
*A0A480Y2E3_PIG *Kininogen-1 isoform 1*	*Sus scrofa* Wild boar	16 (5)	333
*A0A6J0YKX8_ODOVR *Protein AMBP*	*Odocoileus virginianus texanus* White-tailed deer	6 (5)	324
**A0A4W2DA54_BOBOX** *Uncharacterized protein* *(Heparan sulfate proteoglycan 2)*	*Bos indicus x Bos taurus* Zebu x Cow	4 (3)	314
*A0A5J5MM09_MUNRE *Plasminogen*	*Muntiacus reevesi* Chinese muntjac	9 (2)	308
*A0A212C7P2_CEREH *PLG*	*Cervus elaphus hippelaphus* European red deer	8 (2)	299
*A0A5N3WQN5_MUNMU *Vitellogenin domain-containing protein*	*Muntiacus muntjac* Barking deer	8 (1)	278
*A0A5N3X9D4_MUNRE *SERPIN domain-containing protein*	*Muntiacus reevesi* Chinese muntjac	6 (4)	270
*A0A6J0YIK3_ODOVR *Vitamin D-binding protein*	*Odocoileus virginianus texanus* White-tailed deer	5 (4)	267
*A0A6J0XXC2_ODOVR *Apolipoprotein B-100 isoform X1*	*Odocoileus virginianus texanus* White-tailed deer	7 (1)	267
*A0A6J0Y0A8_ODOVR *Serpin A3-7-like*	*Odocoileus virginianus texanus* White-tailed deer	6 (4)	267
*A0A212CS37_CEREH *SERPIN domain-containing protein*	*Cervus elaphus hippelaphus* European red deer	6 (4)	265
**A0A5N3XX47_MUNRE** *Uncharacterized protein (inter-alpha-trypsin inhibitor heavy chain H4)*	*Muntiacus reevesi* Chinese muntjac	5 (3)	259
*A0A6J0VV77_ODOVR *CD5 antigen-like*	*Odocoileus virginianus texanus* White-tailed deer	5 (3)	255
*A0A5N3WVG9_MUNMU *Apolipoprotein H*	*Muntiacus reevesi* Chinese muntjac	4 (3)	252
**A0A4W2E1T0_BOBOX** *Uncharacterized protein* *(FZ domain-containing protein; collagen type XVIII alpha 1 chain; COL18A1 protein)*	*Bos indicus x Bos taurus* Zebu x Cow	5 (3)	244
**A0A2Y9N2V9_DELLE** *Bradykinin*	*Delphinapterus leucas* Beluga whale	19 (5)	235
*A0A212D5I5_CEREH *DSP*	*Cervus elaphus hippelaphus* European red deer	6 (1)	207
**A0A4U1EJD5_MONMO** *TAF domain-containing protein*	*Monodon monoceros* Narwhale	5 (3)	204
*A0A6B0SDR2_9CETA *Glyceraldehyde-3-phosphate dehydrogenase*	*Bos mutus* Wild yak	6 (2)	202
*A0A5N3V0U6_MUNMU *Peptidase_M14 domain-containing protein*	*Muntiacus muntjac* Barking deer	5 (1)	200
**A0A5N3VBS8_MUNMU** *Uncharacterized protein (insulin-like growth factor-binding protein complex acid labile subunit)*	*Muntiacus muntjac* Barking deer	4 (2)	199
**A0A287AAL6_PIG** *Uncharacterized protein* *(four and a half LIM domains protein 1 isoform X3)*	*Sus scrofa* Wild boar	4 (2)	198
*A0A2U4C7Y7_TURTR *Histidine-rich glycoprotein*	*Tursiops truncates* Common bottlenose dolphin	24 (2)	186
**A0A3Q1M1M7_BOVIN** *Junction plakoglobin*	*Bos taurus* Cow	4 (3)	186
**A0A212CSZ9_CEREH** *Ig-like domain-containing protein*	*Cervus elaphus hippelaphus* European red deer	3 (1)	181
*A0A452FXZ3_CAPHI *Apolipoprotein H*	*Capra hircus* Goat	3 (2)	180
*A0A5N3WZL8_MUNMU *Complement C1q subcomponent subunit A*	*Muntiacus muntjac* Barking deer	4 (3)	179
*A0A6J0XZP9_ODOVR *Alpha-1-antitrypsin*	*Odocoileus virginianus texanus*	5 (2)	179
*A0A452E7A0_CAPHI *Plasminogen*	*Capra hircus* Goat	5 (2)	176
**A0A4V5P683_MONMO** *Uncharacterized protein* *(histone H2B type 1-L-like)*	*Monodon monoceros* Narwhale	4 (2)	174
**A0A6B9SCH7_BOVIN** *Ig lamda chain variable region*	*Bos taurus* Cow	2 (2)	160
**A0A452E8D3_CAPHI** *Ig-like domain-containing protein*	*Capra hircus* Goat	3 (2)	157
*A0A452F014_CAPHI *SERPIN domain-containing protein*	*Capra hircus* Goat	4 (1)	155
*A0A2Y9LVH2_DELLE *Amine oxidase*	*Delphinapterus leucas* Beluga whale	2 (2)	141
**A0A6B0SAT2_9CETA** *Ig-like domain-containing protein*	*Bos mutus* Wild yak	4 (1)	140
**A0A4W2CFX9_BOBOX** *Ig-like domain-containing protein*	*Bos indicus x Bos taurus* Zebu x Cow	2 (1)	139
**A2P2I1_SHEEP** *VH region*	*Ovis aries* *Sheep*	2 (1)	139
*A0A088Q0F1_9CETA *Heat shock protein 90kDa alpha*	*Bos grunniens x Bos taurus* Domestic yak x Cow	2 (2)	137
**A0A5N3UK72_MUNRE** *Ig-like domain-containing protein*	*Muntiacus reevesi* Chinese muntjac	2 (1)	133
**FIBA_ALCAA** *Fibrinogen alpha chain*	*Alces alces alces* Moose	1 (1)	122
**A0A643C7L4_BALPH** *Uncharacterized protein (desmoplakin)*	*Balaenoptera physalus* Fin whale	4 (1)	120
**A6QM09_BOVIN** *Uncharacterized protein (Ig-like domain-containing protein; Ig lambda chain V-III region LOI-like protein)*	*Bos taurus* Cow	4 (1)	118
*A0A6B0R457_9CETA *Activating signal cointegrator 1 complex subunit 3*	*Bos mutus* Wild yak	3 (1)	118
*A0A212DB97_CEREH *SERPINF2*	*Cervus elaphus hippelaphus* European red deer	2 (1)	117
*A0A6J3PT56_TURTR *Immunoglobulin lambda-1 light chain-like isoform X1*	*Tursiops truncates* Common bottlenose dolphin	4 (1)	116
**A0A3Q1LT19_BOVIN** *Ig-like domain-containing protein*	*Bos taurus* Cow	2 (2)	113
*A0A212CSZ1_CEREH *SERPINA5*	*Cervus elaphus hippelaphus* European red deer	3 (1)	112
**A0A5N4CT25_CAMDR** *Histone H4*	*Camelus dromedarius* Dromedary	3 (1)	107
*A0A6J0XAN1_ODOVR *Complement component C9*	*Odocoileus virginianus texanus* White-tailed deer	3 (1)	104
*A0A6J0Y2I3_ODOVR *Alpha-1B-glycoprotein*	*Odocoileus virginianus texanus* White-tailed deer	2 (1)	95
**A0A2F0AYU0_ESCRO** *Ig lambda chain V-III region SH*	*Eschrichtius robustus* Gray whale	2 (1)	95
**A0A212CS30_CEREH** *Ig-like domain-containing protein*	*Cervus elaphus hippelaphus* European red deer	2 (1)	93
*A0A1L6BNZ0_BUBBU *Alpha-S1-casein*	*Bubalus bubalis* Water buffalo	2 (1)	93
*A0A212D4C7_CEREH *Ribosomal protein*	*Cervus elaphus hippelaphus* European red deer	1 (1)	92
*A0A212CIC4_CEREH *FETUB*	*Cervus elaphus hippelaphus* European red deer	2 (1)	91
**A0A0R4I993_SUSBA** *Tubulin alpha chain*	*Sus barbatus* Bornean bearded pig	2 (1)	89
**A2P2I3_SHEEP** *VH region*	*Ovis aries* Sheep	2 (1)	89
*A0A6J0WSX6_ODOVR *Tubulin beta-3 chain*	*Odocoileus virginianus texanus* White-tailed deer	2 (1)	88
*A0A340WKS1_LIPVE *Selenoprotein P*	*Lipotes vexillifer* Baiji	3 (1)	87
*A0A4W2BXS4_BOBOX *Kallikrein B1*	*Bos indicus x Bos taurus* Zebu x Cow	2 (1)	86
**R4R2H5_SHEEP** *Beta-casein*	*Ovis aries* Sheep	2 (1)	84
*A0A6J0Z7P6_ODOVR *Apolipoprotein R-like*	*Odocoileus virginianus texanus* White-tailed deer	2 (1)	84
**A0A6B9SDT6_BOVIN** *Ig lamda chain variable region*	*Bos taurus* Cow	1 (1)	84
*A0A5N3WWG2_MUNMU *SERPIN domain-containing protein*	*Muntiacus muntjac* Barking deer	2 (1)	83
*A0A2Y9EXF5_PHYMC *2-phospho-D-glycerate hydro-lyase*	*Physeter macrocephalus* Sperm whale	1 (1)	82
*A0A212D4I5_CEREH *C3/C5 convertase*	*Cervus elaphus hippelaphus* European red deer	3 (1)	81
*A0A212CM12_CEREH *40S ribosomal protein S18*	*Cervus elaphus hippelaphus* European red deer	1 (1)	80
**A0A452G1G8_CAPHI** *Uncharacterized protein (msx2-interacting protein isoform X, X2, X3, X4)*	*Capra hircus* Goat	3 (1)	79
**A0A212CAL2_CEREH** *Elongation factor 1-alpha*	*Cervus elaphus hippelaphus* European red deer	2 (2)	79
*A0A212CI11_CEREH *Alpha-2-HS-glycoprotein*	*Cervus elaphus hippelaphus* European red deer	1 (1)	78
**A0A2F0B9E6_ESCRO** *Trypsin*	*Eschrichtius robustus* Gray whale	5 (2)	78
*A0A5J5MZJ4_MUNRE *MACPF domain-containing protein*	*Muntiacus reevesi* Chinese muntjac	2 (1)	74
*A0A212C6Y8_CEREH *Transthyretin*	*Cervus elaphus hippelaphus* European red deer	1 (1)	73
*A0A212D5R7_CEREH *JCHAIN*	*Cervus elaphus hippelaphus* European red deer	2 (1)	73
*A0A480MMJ7_PIG *Heat shock 70 kDa protein*	*Sus scrofa* Wild boar	2 (2)	72
*A0A6J0Y8N1_ODOVR *Angiopoietin-related protein 6 isoform X2*	*Odocoileus virginianus texanus* White-tailed deer	2 (1)	72
*A0A5N4EH44_CAMDR *Biorientation of chromosomes in cell division protein 1-like 1*	*Camelus dromedarius* Dromedary	2 (1)	71
*S9WER1_CAMFR *Biorientation of chromosomes in cell division protein 1-like protein*	*Camelus ferus* Wild Bactrian camel	2 (1)	71
**A0A212D1P2_CEREH** *Uncharacterized protein (N-acetylmuramoyl-L-alanine amidase)*	*Cervus elaphus hippelaphus* European red deer	2 (1)	70
*A0A3Q1LUP1_BOVIN *Uncharacterized protein* *(cilia- and flagella-associated protein 54)*	*Bos taurus* Cow	3 (1)	69
**A0A212CM59_CEREH** *Ig-like domain-containing protein*	*Cervus elaphus hippelaphus* European red deer	4 (1)	68
*A0A2Y9EUI8_PHYMC *Arachidonate 15-lipoxygenase*	*Physeter macrocephalus* Sperm whale	3 (1)	67
**A2P2H1_SHEEP** *VH region*	*Ovis aries* Sheep	2 (1)	66
*A0A0B8RZA9_PIG *Proliferation-associated 2G4, 38kDa)*	*Sus scrofa* Wild boar	2 (2)	66
**A0A4X1TXJ2_PIG** *Uncharacterized protein (IgG heavy chian constant region)*	*Sus scrofa* Wild boar	2 (1)	63
**A0A2Y9EH04_PHYMC** *Fer-1-like protein 4*	*Physeter macrocephalus* Sperm whale	3 (1)	62
*A0A286ZRK7_PIG *60S ribosomal protein L11*	*Sus scrofa* Wild boar	1 (1)	62
**A0A4W2CHE4_BOBOX** *IF rod domain-containing protein*	*Bos indicus x Bos taurus* Zebu x Cow	3 (1)	61
*A0A287BDT6_PIG *Ubiquitin carboxyl-terminal hydrolase*	*Sus scrofa* Wild boar	2 (1)	61
*A0A287AFA5_PIG *Endoplasmin*	*Sus scrofa* Wild boar	1 (1)	61
*BIP_BOVIN *Endoplasmic reticulum chaperone BiP*	*Bos taurus* Cow	1 (1)	58
**0A6B0R269_9CETA** *Ig-like domain-containing protein*	*Bos mutus* Wild yak	1 (1)	57
**A0A5N3UHJ8_MUNRE** *Ig-like domain-containing protein*	*Muntiacus reevesi* Chinese muntjac	1 (1)	56
*A0A2Y9M486_DELLE *Protein PRRC2C isoform X8*	*Delphinapterus leucas* Beluga whale	9 (1)	55
*A0A383Z8A9_BALAS *Putative SEC14-like protein 6*	*Balaenoptera acutorostrata scammony* Minke whale	2 (1)	53
*A0A212D225_CEREH *TMED9*	*Cervus elaphus hippelaphus* European red deer	1 (1)	53
*A0A0B8RSX6_PIG *Filamin A, alpha*	*Sus scrofa* Wild boar	2 (1)	52
*A0A452E6D4_CAPHI *Complement C5-like*	*Capra hircus* Goat	2 (1)	52
**A0A6B0RW97_9CETA** *Uncharacterized protein* *(Ig lamda chain variable region)*	*Bos mutus* Wild yak	1 (1)	52
*A0A212CJY0_CEREH *Transferrin receptor protein 1*	*Cervus elaphus hippelaphus* European red deer	1 (1)	52
*A0A4W2F326_BOBOX *Anaphylatoxin-like domain-containing protein*	*Bos indicus x Bos taurus* Zebu x Cow	2 (1)	52
**A0A4U1EAQ3_MONMO** *Ig-like domain-containing protein*	*Monodon monoceros* Narwhal	2 (1)	50
**A0A286ZJV6_PIG** *Annexin*	*Sus scrofa* Wild boar	1 (1)	50
**A0A6B0RTH8_9CETA** *Uncharacterized protein* *(obscurin, cytoskeletal calmodulin and titin-interacting RhoGEF)*	*Bos mutus* Wild yak	2 (1)	50
*A0A212CKA1_CEREH *Peptidyl-prolyl cis-trans isomerase*	*Cervus elaphus hippelaphus* European red deer	1 (1)	50
**W5P6D4_SHEEP** *Uncharacterized protein* *(Integrator complex subunit 1)*	*Ovis aries* Sheep	2 (1)	50
*A0A4W2F827_BOBOX *60 kDa poly(U)-binding-splicing factor*	*Bos indicus x Bos taurus* Zebu x Cow	3 (1)	50
*A0A212CT53_CEREH *Lactadherin*	*Cervus elaphus hippelaphus* European red deer	2 (1)	50
**A0A6B9SDX6_BOVIN** *Ig lamda chain variable region*	*Bos taurus* Cow	1 (1)	50

† Ions score is −10*Log(P), where P is the probability that the observed match is a random event. Individual ions scores > 46 indicate identity or extensive homology (*p* < 0.05). Protein scores are derived from ions scores as a non-probabilistic basis for ranking protein hits.

## Data Availability

Data are contained within the article and supplementary material.

## Supplementary Materials

The following are available online at https://www.mdpi.com/2079-7737/10/3/222/s1, Table S1: Full LC–MS/MS analysis of F95-enriched proteins identified in reindeer *(R. tarandus)* plasma EVs. Table S2: Full LC–MS/MS analysis of F95-enriched proteins identified in reindeer *(R. tarandus)* whole plasma. Figure S1: A. Biological processes GO pathways for STRING protein networks of deiminated proteins in reindeer *(R. tarandus)* plasma EVs. B. Molecular function GO pathways for STRING protein networks of deiminated proteins in plasma EVs. Figure S2: A. Biological processes GO pathways for STRING protein networks of deiminated proteins in reindeer *(R. tarandus)* whole plasma. B. Molecular function GO pathways for STRING protein networks of deiminated proteins in reindeer *(R. tarandus)* whole plasma.
