# Supplementary figures and images for: Post-Translational Protein Deimination Signatures in Plasma and Plasma EVs of Reindeer (Rangifer tarandus)
Source: Biology (Basel). 2021 Mar 13;10(3):222. doi: 10.3390/biology10030222 (PMC7998281; doi:10.3390/biology10030222)

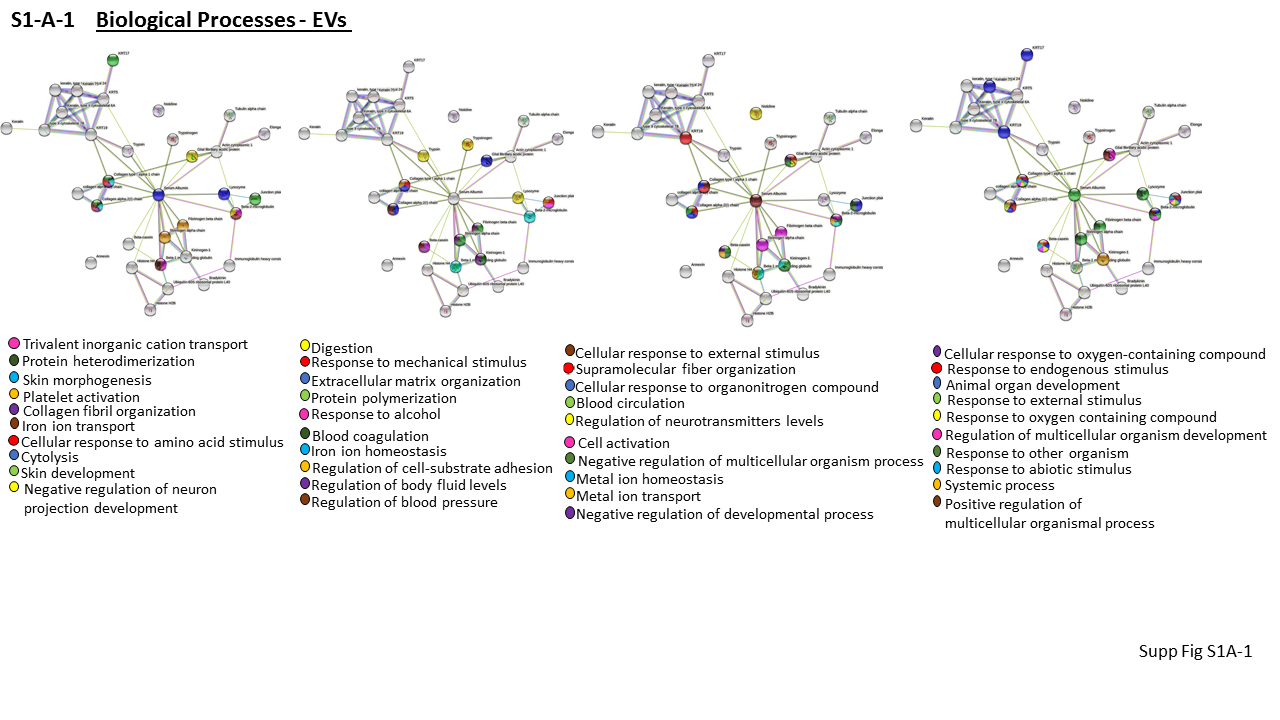

Supplement: Supplementary file 1 [file biology-10-00222-s001.zip › Supp Fig S1 A-1.tif]

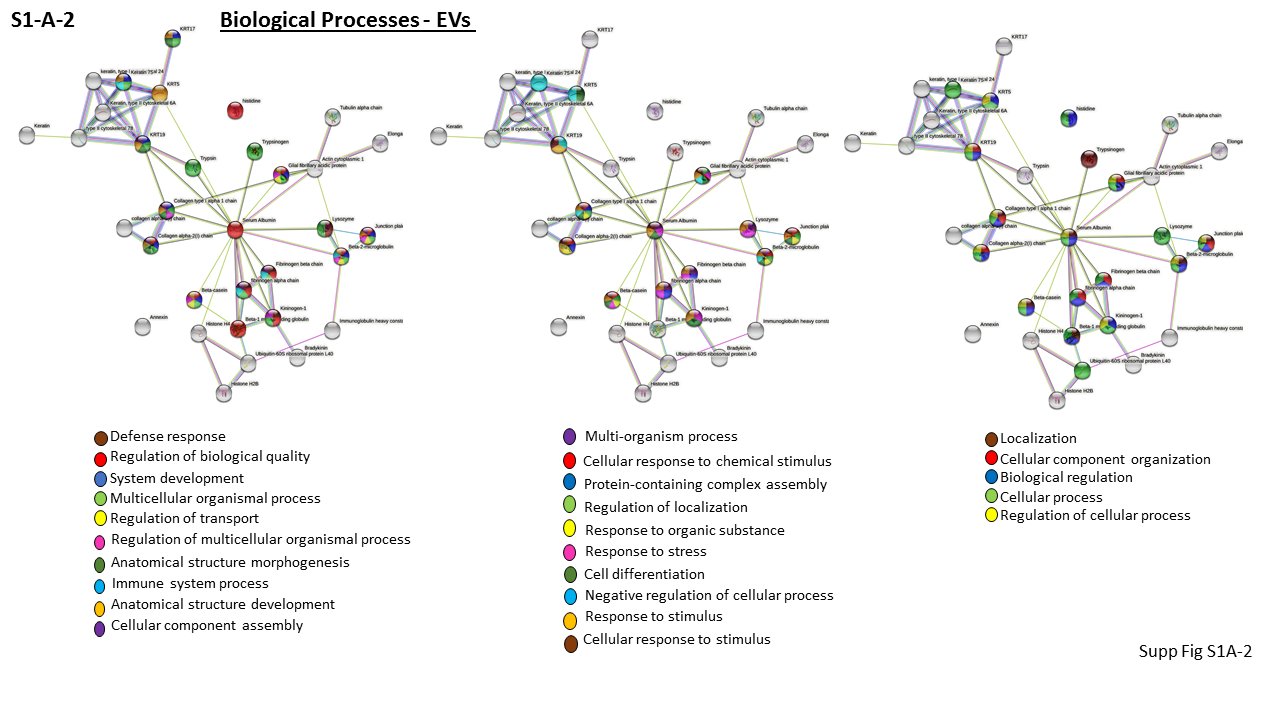

Supplement: Supplementary file 1 [file biology-10-00222-s001.zip › Supp Fig S1 A-2.tif]

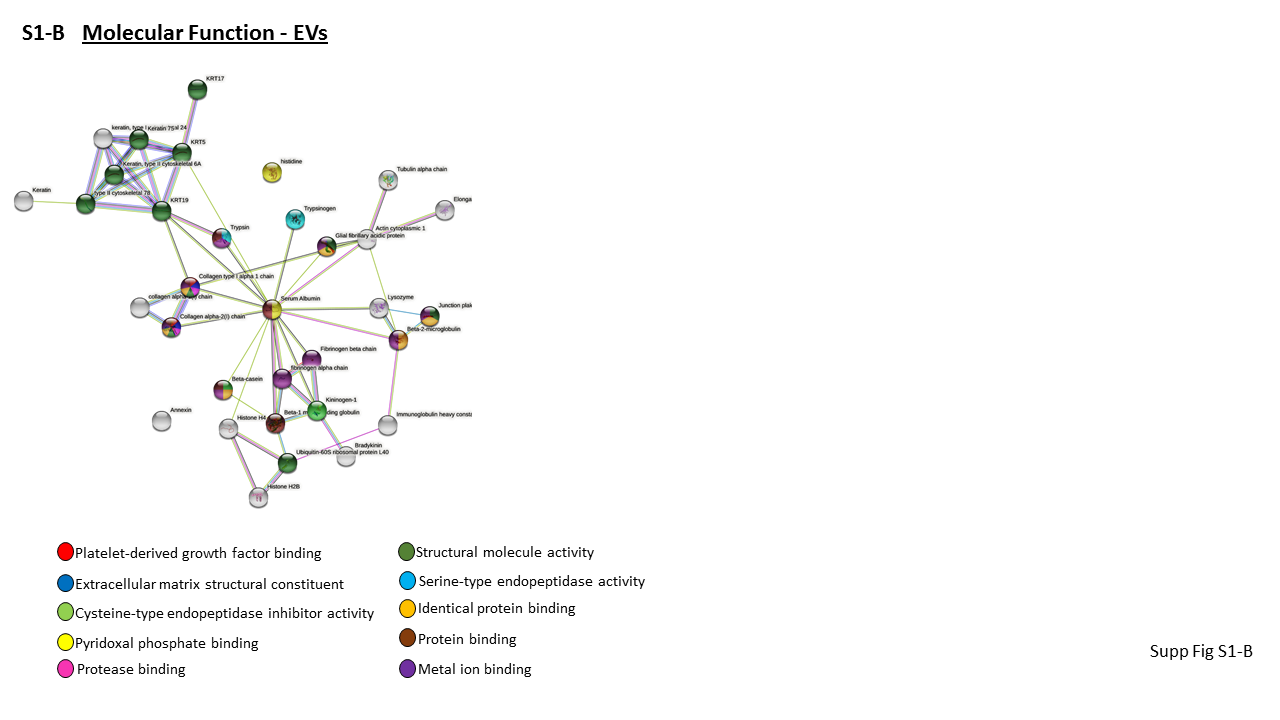

Supplement: Supplementary file 1 [file biology-10-00222-s001.zip › Supp Fig S1 B.tif]

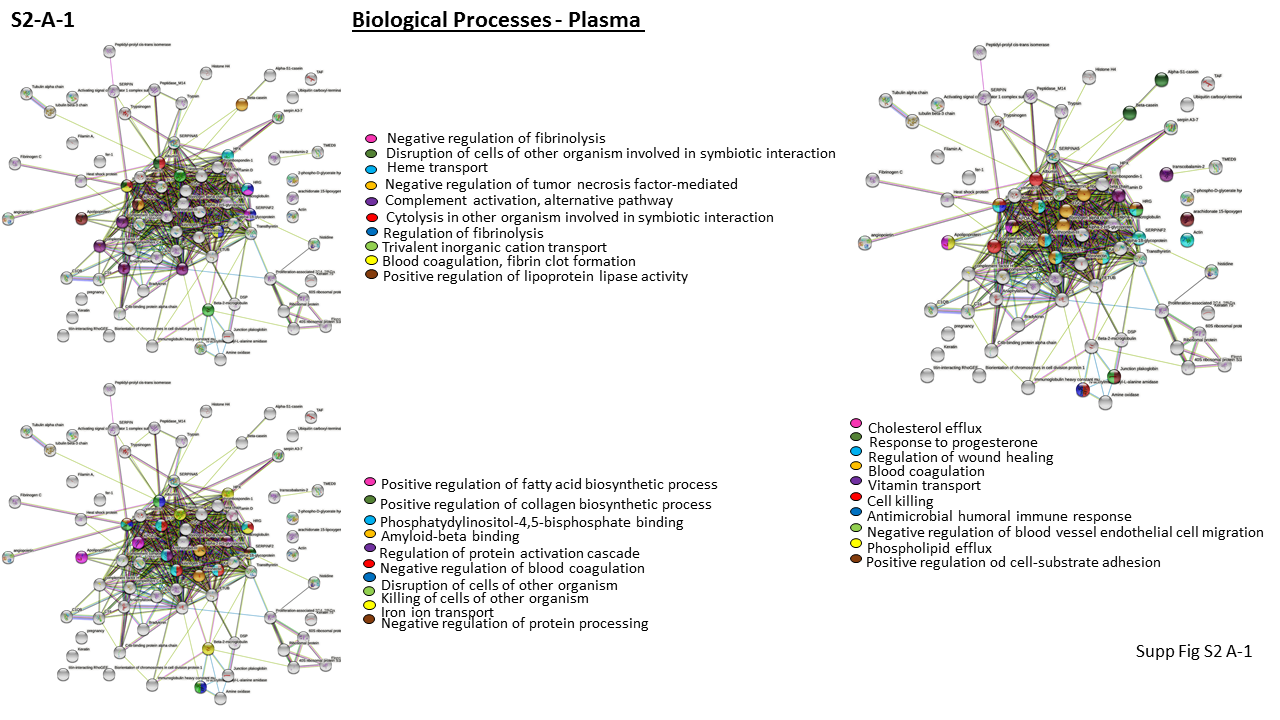

Supplement: Supplementary file 1 [file biology-10-00222-s001.zip › Supp Fig S2 A-1.tif]

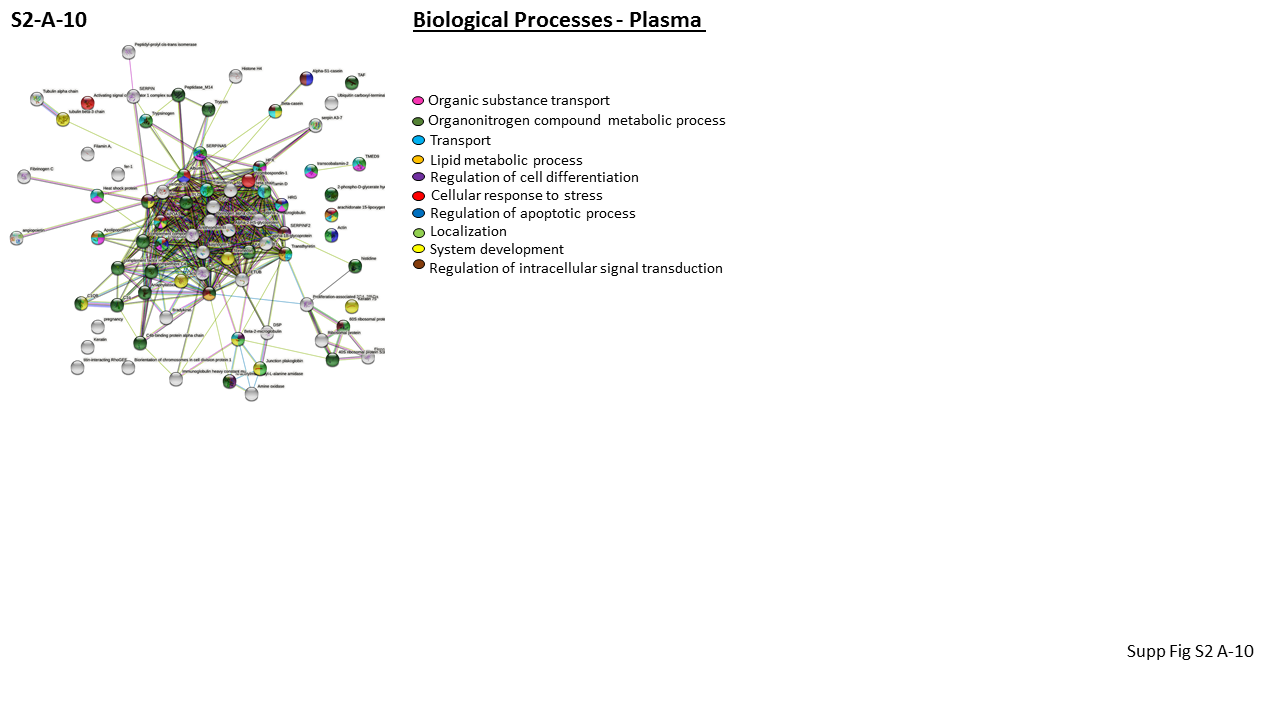

Supplement: Supplementary file 1 [file biology-10-00222-s001.zip › Supp Fig S2 A-10.tif]

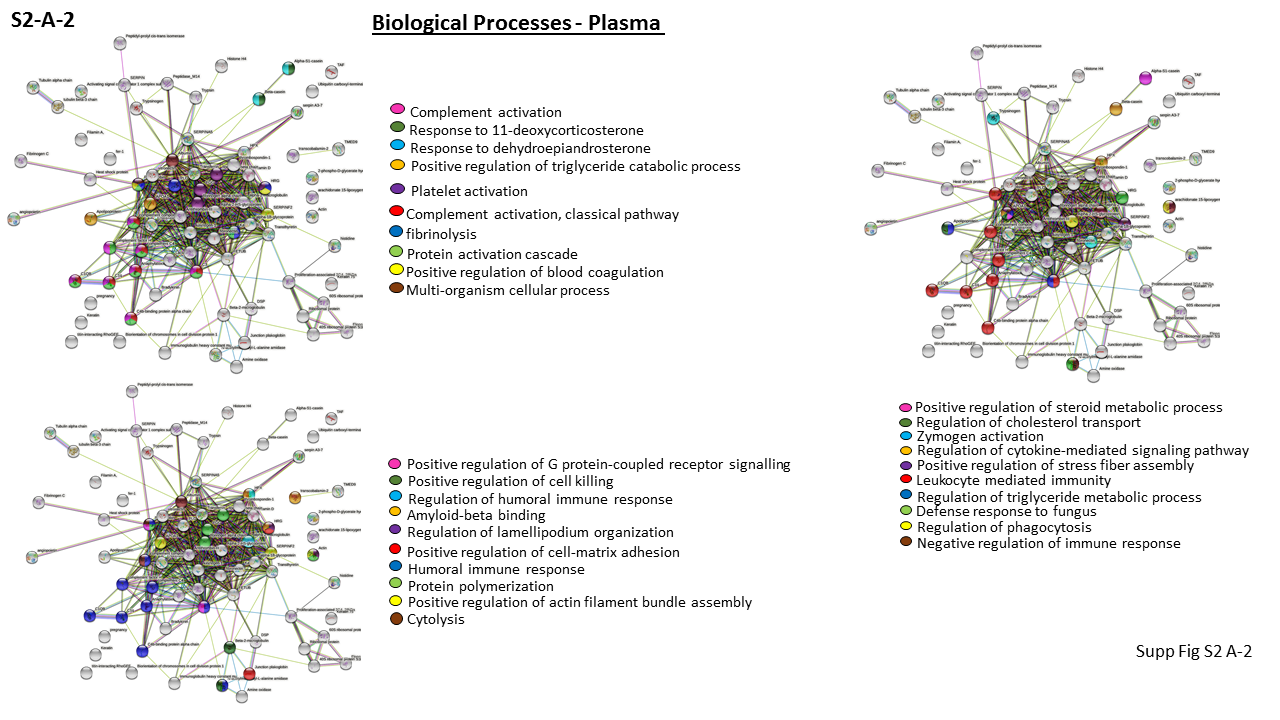

Supplement: Supplementary file 1 [file biology-10-00222-s001.zip › Supp Fig S2 A-2.tif]

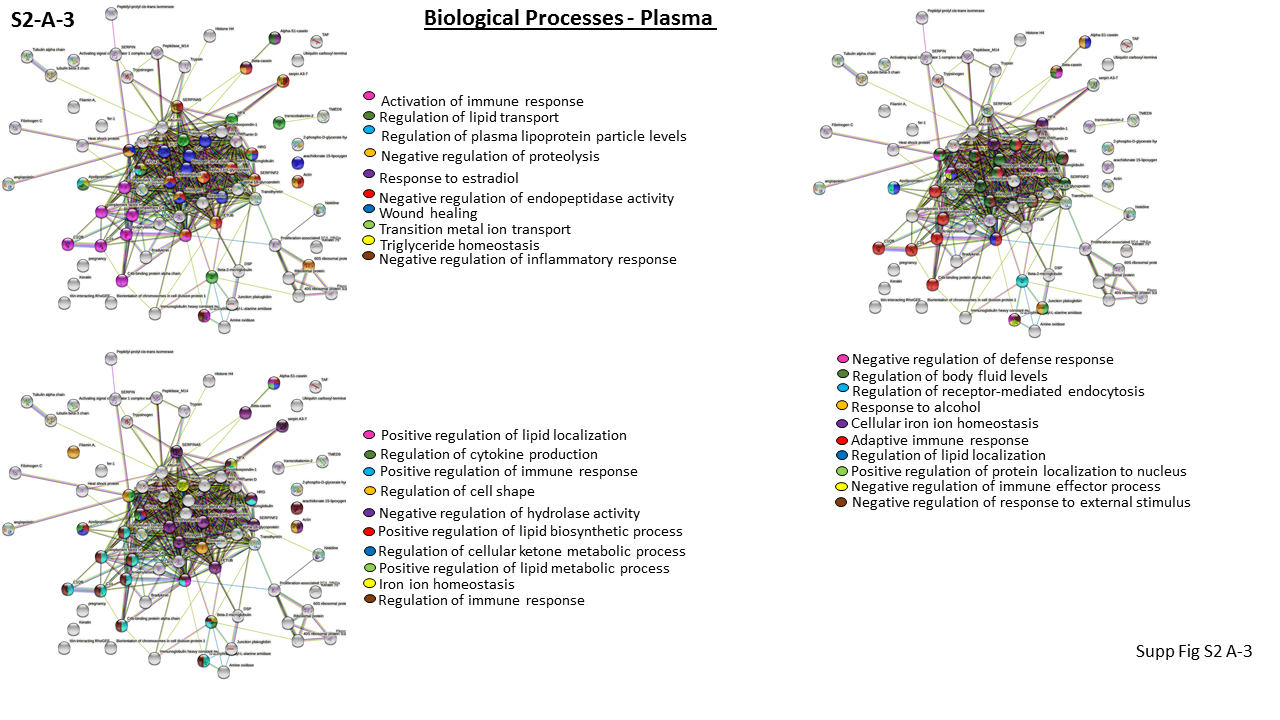

Supplement: Supplementary file 1 [file biology-10-00222-s001.zip › Supp Fig S2 A-3.tif]

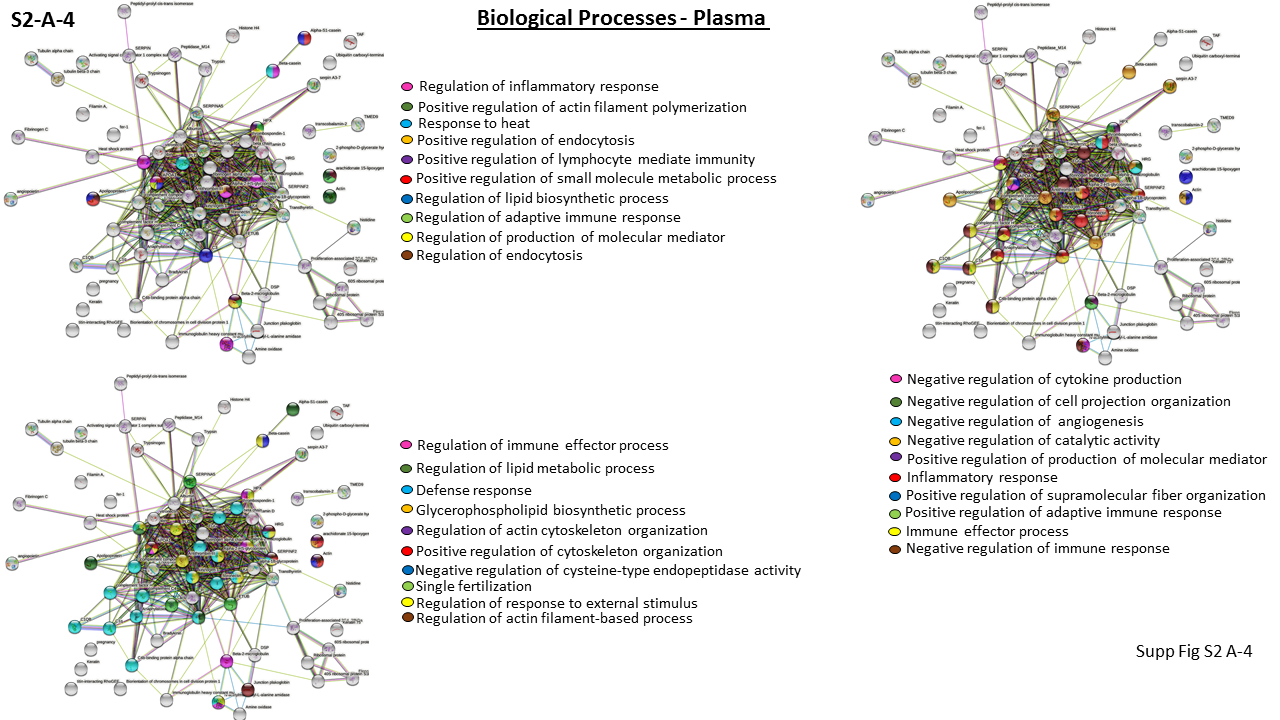

Supplement: Supplementary file 1 [file biology-10-00222-s001.zip › Supp Fig S2 A-4.tif]

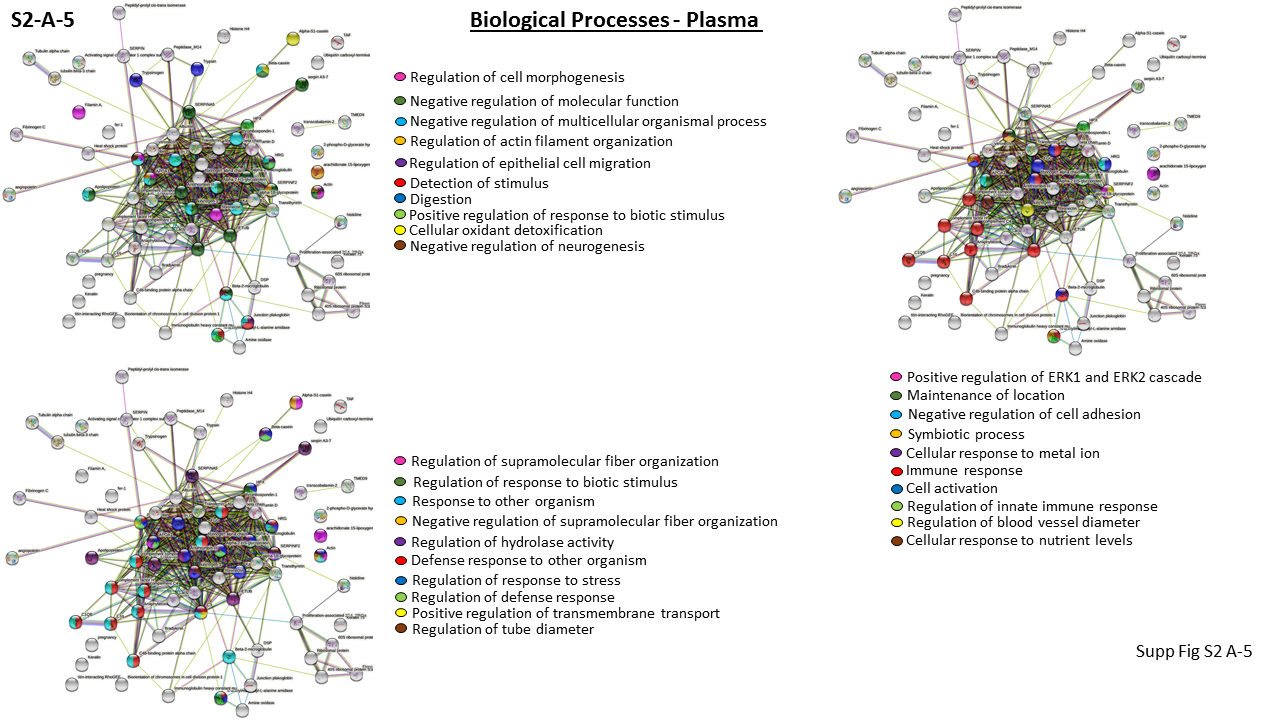

Supplement: Supplementary file 1 [file biology-10-00222-s001.zip › Supp Fig S2 A-5.tif]

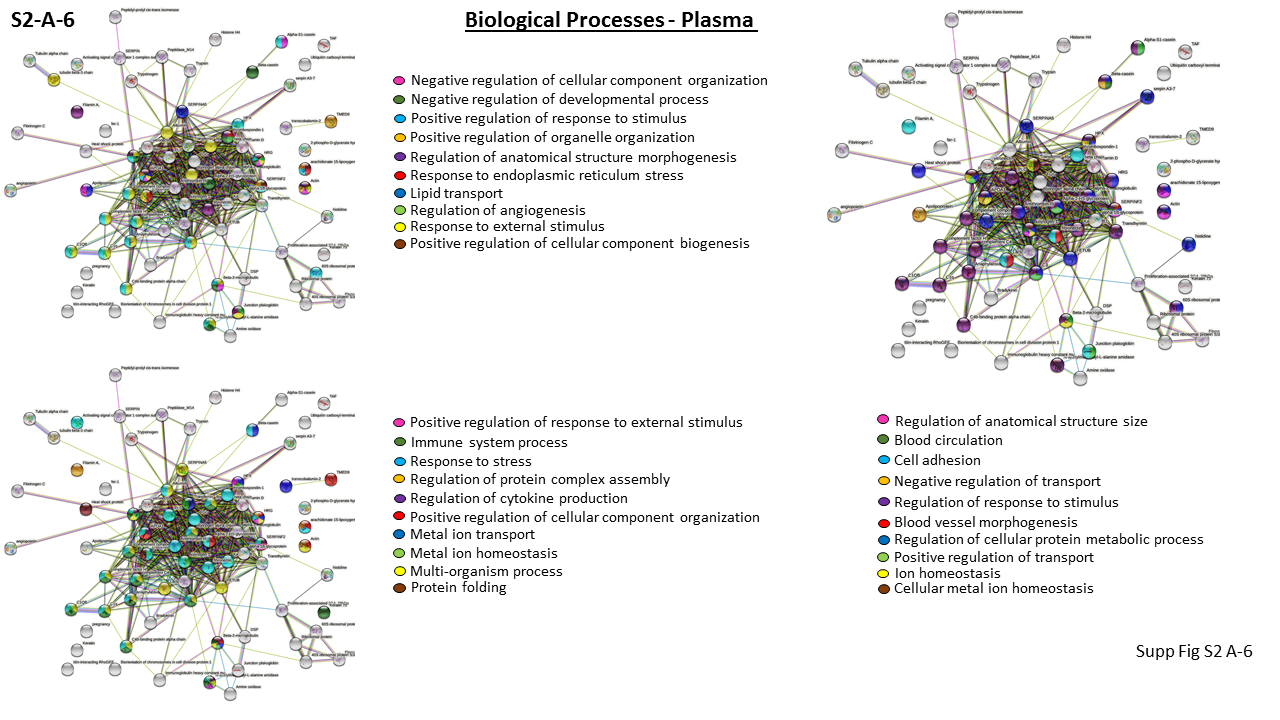

Supplement: Supplementary file 1 [file biology-10-00222-s001.zip › Supp Fig S2 A-6.tif]

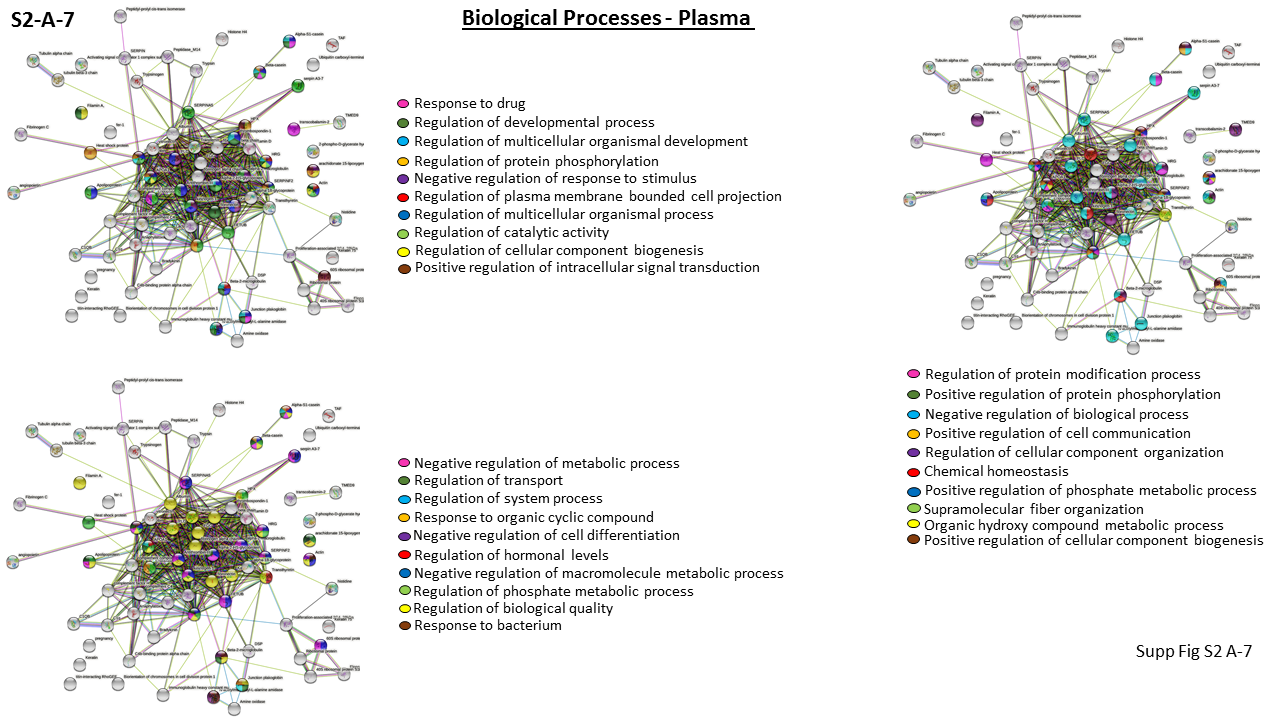

Supplement: Supplementary file 1 [file biology-10-00222-s001.zip › Supp Fig S2 A-7.tif]

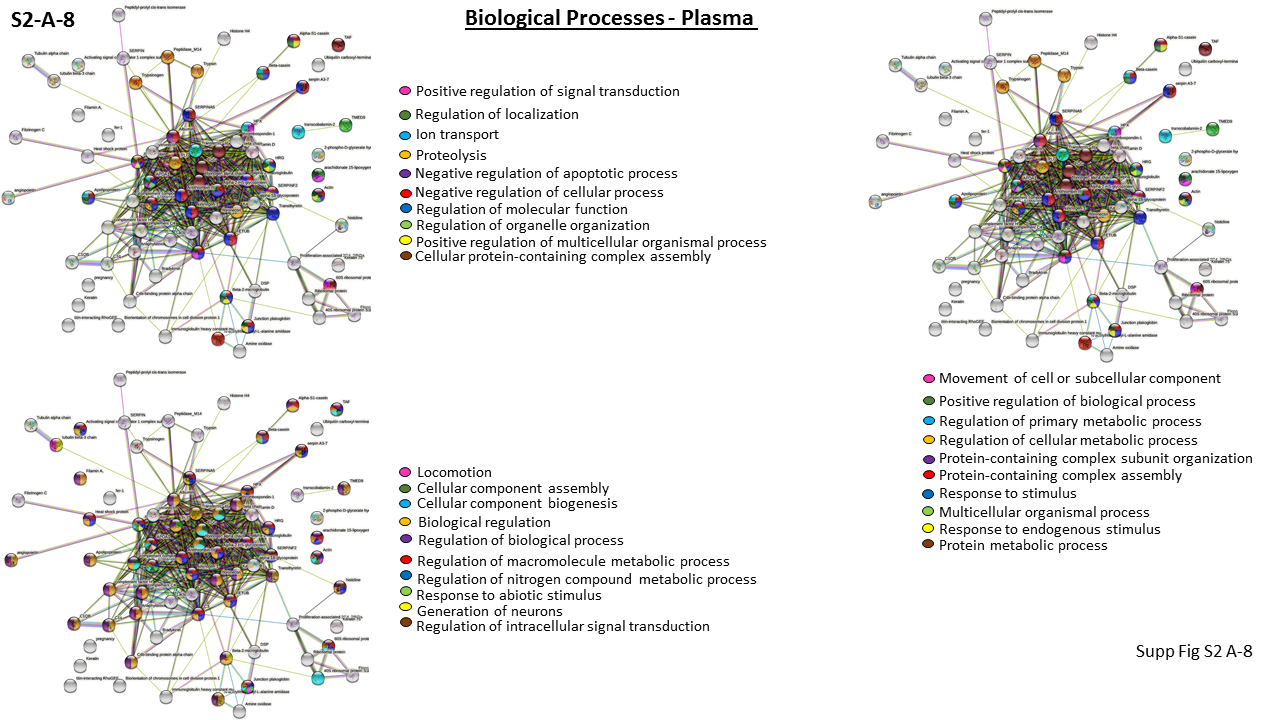

Supplement: Supplementary file 1 [file biology-10-00222-s001.zip › Supp Fig S2 A-8.tif]

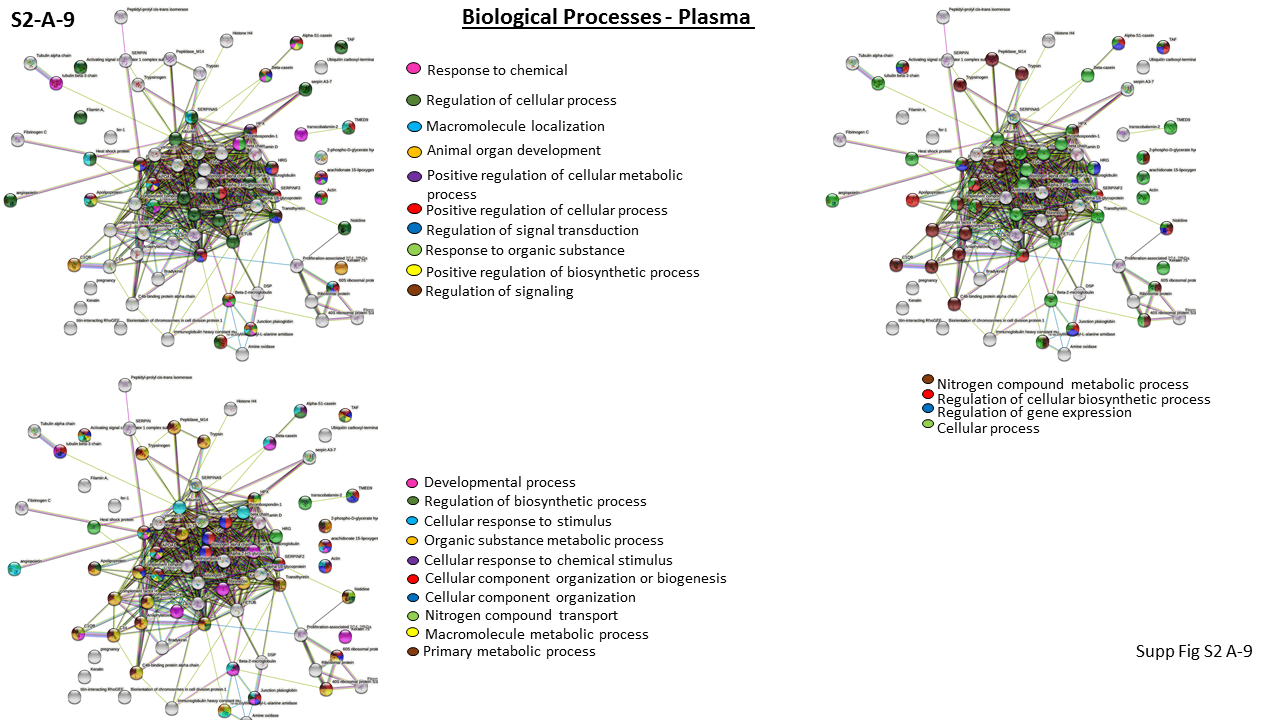

Supplement: Supplementary file 1 [file biology-10-00222-s001.zip › Supp Fig S2 A-9.tif]

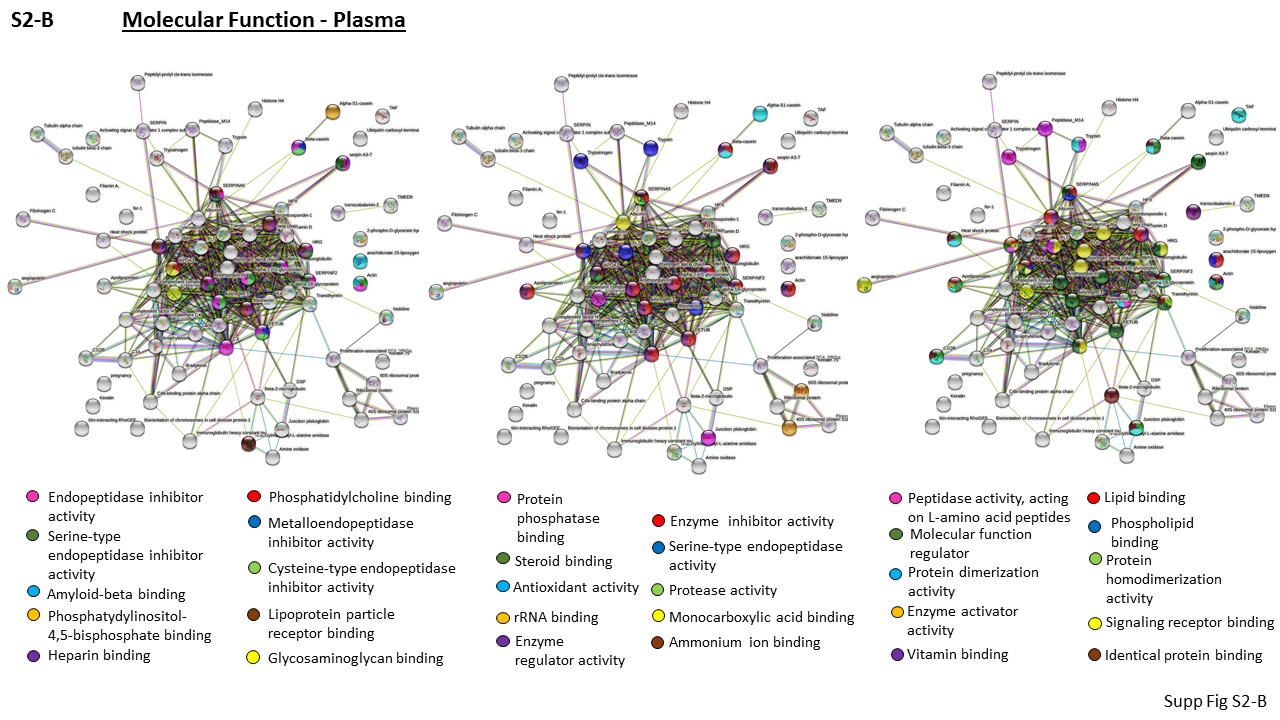

Supplement: Supplementary file 1 [file biology-10-00222-s001.zip › Supp Fig S2 B.tif]

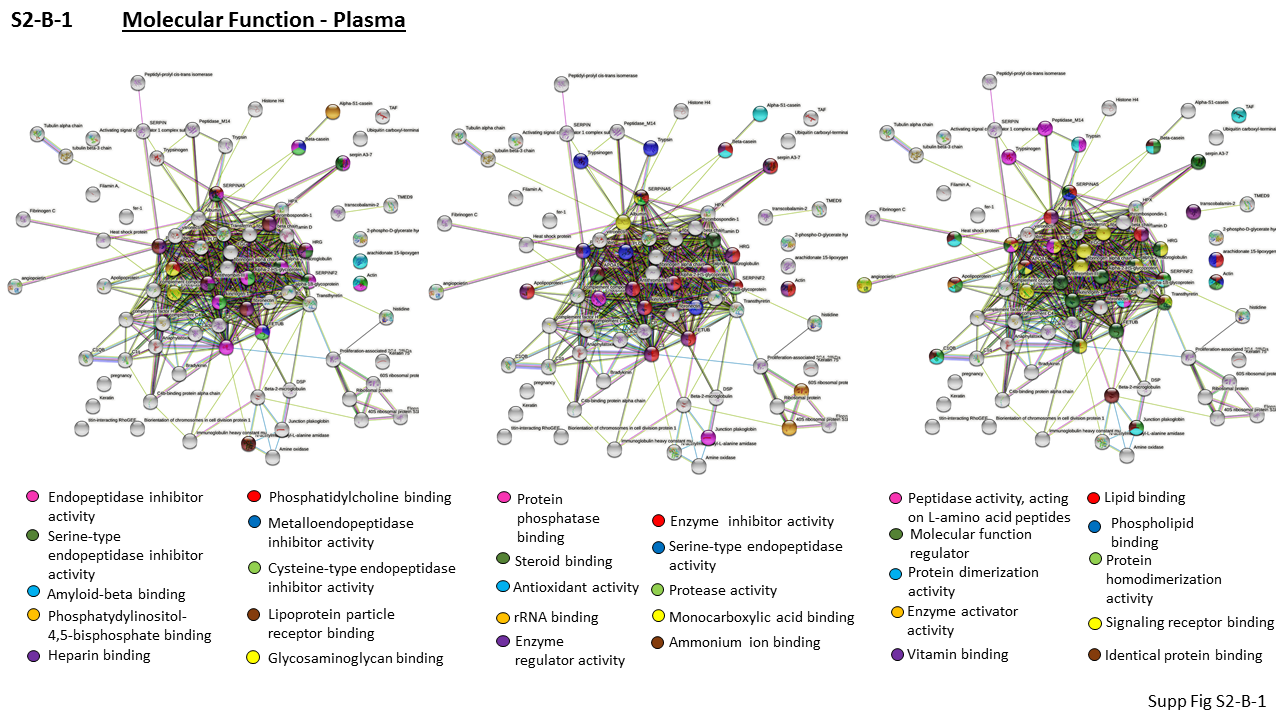

Supplement: Supplementary file 1 [file biology-10-00222-s001.zip › Supp Fig S2 B-1.tif]

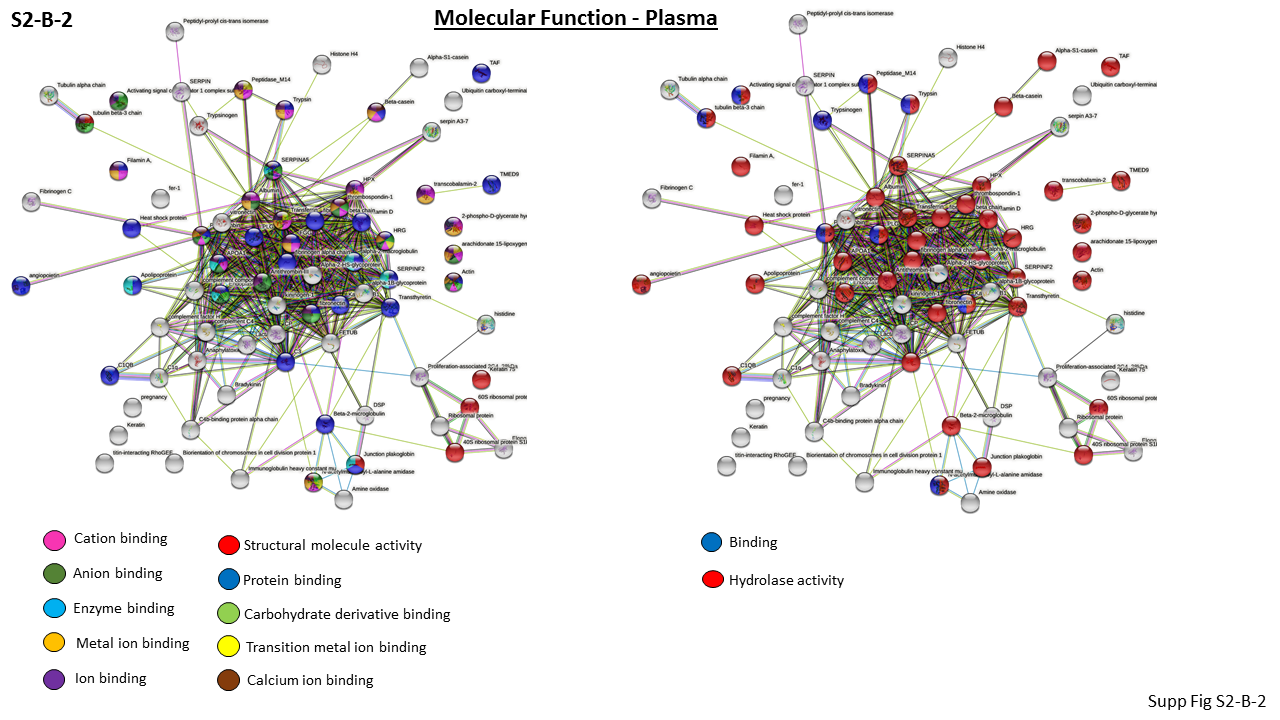

Supplement: Supplementary file 1 [file biology-10-00222-s001.zip › Supp Fig S2 B-2.tif]
